# Operative Indications for Lateral Compression Type 1 Pelvic Ring Injuries: A Systematic Review of Reported Selection Criteria

**DOI:** 10.3390/jcm15207891

**Published:** 2026-10-13

**Authors:** Jaeheon Lee, Hyung Keun Song

**Affiliations:** 1 Department of Orthopedic Surgery, Saeson Hospital, Daejeon 34908, Republic of Korea; leejaeheon.kr@gmail.com; 2 Department of Medical Sciences, Graduate School of Ajou University, Suwon 16499, Republic of Korea; 3 Department of Orthopaedic Surgery, Ajou University School of Medicine, Suwon 16499, Republic of Korea

**Keywords:** lateral compression, LC-1, pelvic ring injury, operative indication, treatment selection, examination under anesthesia, stress radiography, systematic review, reporting standards

## Abstract

**Background/Objectives**: Lateral compression type 1 (LC-1) injuries range from stable patterns to injuries whose instability is not evident on initial imaging. How published studies define the criteria for operative fixation has not been characterized. **Methods**: We systematically reviewed studies reporting operative-selection criteria for LC-1 injuries. MEDLINE and Embase were searched (last update 14 August 2026) without date or language filters; English-language full text was required for eligibility, and dedicated fragility-fracture cohorts were excluded. Reports of the same or nested cohorts were linked into study-level units. Each unit was coded for indication category, assessment method, quantitative threshold, and selection-rule reconstructibility (whether the assessment, positive rule, and link to the operation could be reconstructed). A second clinician independently recoded three of these fields for all units. Registration (retrospective): OSF, 10.17605/OSF.IO/WSDGX. **Results**: Thirty-seven eligible reports contributed 29 study-level units. Selection was multifactorial in 15 units (51.7%), 16 (55.2%) used dynamic or stress assessment, and 18 (62.1%) reported a quantitative threshold, most often approximately 1 cm (10 units; 7 after collapsing four units from one institutional registry). Only 6 units (20.7%) met the prespecified definition of a fully reconstructible operative-selection rule. Of the remaining 23, two, by design, had no ascertainable clinical selection pathway; the other 21 all lacked a reconstructible positive rule (14 units), a clear criterion-to-treatment linkage (16), or both, whereas the assessment itself was non-reconstructible in only two. Inter-coder agreement for selection-rule reconstructibility was 89.7% (26/29). **Conclusions**: Operative indications for LC-1 injuries remain heterogeneous and incompletely operationalized. What to assess was almost always identifiable; reconstruction failed after it. Studies should report how instability is elicited, how a positive test is defined, and what that result caused.

## 1. Introduction

Lateral compression type 1 (LC-1) injuries are among the most common patterns of pelvic ring injury, but the designation does not describe a single mechanical or clinical entity. It encompasses injuries that remain stable with nonoperative care as well as injuries in which clinically relevant instability is not apparent on presentation radiographs or computed tomography. Treatment selection is therefore consequential in both directions: occult instability may lead to displacement or persistent functional limitation if not recognized, whereas fixation of an injury that would have healed uneventfully exposes the patient to operative and anesthetic risk without corresponding benefit. The central clinical question is not whether LC-1 injuries can be treated nonoperatively, but how to identify the subset for whom fixation is justified.

Because static imaging may not disclose the full extent of instability, dynamic assessment has been developed as an adjunct. Examination under anesthesia with fluoroscopic stress views was introduced to reveal displacement not evident on presentation imaging [1], and awake techniques subsequently included lateral stress radiography [2], emergency-department stress radiography [3], pelvic-binder radiography [4], and stress ultrasonography [5]. Yet improved characterization of mechanical stability has not fully resolved the treatment decision. In a surgeon survey, adding examination-under-anesthesia findings substantially improved agreement about whether the pelvic ring was mechanically stable, while agreement about the need for surgery improved far less [6]. Separately, established pelvic-ring classifications [7,8] have shown only moderate interobserver agreement [9], and a large survey of Orthopaedic Trauma Association members found only fair agreement on whether LC-1 injuries should be treated operatively [10]. These observations suggest that uncertainty persists not only in how instability is assessed, but also in how assessment findings are translated into treatment.

Previous evidence syntheses have addressed different parts of this problem. Varma et al. [11] compared operative with nonoperative treatment in non-fragility LC-1 injuries with complete sacral fractures; only five observational studies met their criteria, and formal meta-analysis was not possible. Importantly, the authors noted that eligibility criteria for surgery varied across studies, with some emphasizing pain and mobility and others initial displacement on static radiographs. Timmer et al. [12] reviewed operative indications in elderly patients with low-energy pelvic ring fractures and found no generally accepted criteria for fixation in that population; persistent pain was the most frequently reported indication, while stability-based fracture classification appeared to be of secondary importance. More recently, Burapachaisri et al. [13] reviewed the evidence supporting pelvic stress radiographs and their role in identifying patients for surgery, providing a focused synthesis of dynamic stress assessment rather than of operative selection as a whole.

Taken together, these reviews expose different components of the same upstream problem: studies do not necessarily define the population considered suitable for surgery in the same way, and a reported finding does not always translate into fixation through an explicit and reproducible rule. This limits not only treatment-effect synthesis but also comparison among diagnostic or stress-assessment strategies, because apparently similar cohorts may have been assembled using different selection pathways. To our knowledge, however, no prior review has systematically treated the operative indication itself—including the assessment used, the positive rule applied, and the linkage from that rule to the treatment actually delivered—as the primary object of synthesis.

We therefore undertook a systematic review of the criteria reported for selecting patients with LC-1 pelvic ring injuries for operative fixation, taking the operative indication itself—whether static and morphological, dynamic, clinical, mixed, or discretionary—as the object of synthesis rather than any single assessment method or patient subgroup. The primary outcome was the type and operational definition of the criteria used to indicate operative fixation, including whether the reported pathway from assessment to treatment could be reconstructed from the publication.

This review is descriptive and methodological in purpose. It does not compare the effectiveness of operative and nonoperative treatment, evaluate the diagnostic accuracy of any stress examination, or propose a new classification of LC-1 instability. Its purpose is to characterize how operative-selection criteria have been reported and operationalized in the published literature.

## 2. Materials and Methods

### 2.1. Review Design and Reporting Framework

This systematic review was designed to identify and characterize the criteria reported for selecting patients with lateral compression type 1 (LC-1) pelvic ring injuries for operative treatment. The review question was: What criteria have been reported for selecting patients with LC-1 pelvic ring injuries for operative treatment? The primary outcome was the type and operational definition of the reported operative-selection criteria.

The review was conducted as a descriptive methodological systematic review. No meta-analysis of treatment effects was planned because the purpose was to map and characterize operative-selection criteria rather than estimate the comparative effectiveness of operative versus nonoperative treatment. Reporting follows PRISMA 2020 [14], with the literature-search methods additionally reported using PRISMA-S [15].

The governing review plan and 11-field extraction codebook were locked on 12 August 2026 before full extraction (LC1-SR-final-plan-v2.2-CODEBOOK-LOCK-2026-08-12.md). Because screening and extraction were completed before public OSF deposit, the OSF record is described as a retrospective protocol/codebook deposit with a dated audit trail, not as prospective preregistration. The review was registered in OSF Registries (registration DOI: 10.17605/OSF.IO/WSDGX; date of registration: 14 August 2026). The original locked plan, as-used search strategies, extraction codebook, reconciliation record, and study-level dataset are available through that registration. The review was not eligible for PROSPERO registration because its outcome is how operative indications are reported rather than a direct health-related outcome, and because data extraction had been completed before registration.

### 2.2. Eligibility Criteria

Studies were eligible when the primary analytic population was explicitly defined as LC-1 or when LC-1-specific treatment-selection information was separately extractable from a broader pelvic-ring cohort. No arbitrary percentage threshold was used to define a “predominantly LC-1” cohort, and contemporary AO/OTA codes [16] were not retrospectively imposed on historical reports.

Dedicated fragility-fracture, low-energy geriatric pelvic-fracture, or elderly low-energy cohorts were excluded. General adult LC-1 cohorts were not excluded solely because they included older patients or ground-level falls.

To be within the treatment-selection sampling frame, a report had to meet at least one of the following conditions: (1) operative and nonoperative LC-1 management were compared or distinguished; (2) an operative eligibility or selection criterion was explicitly reported; (3) an instability, clinical, or radiographic criterion was linked to the treatment decision; or (4) a nonoperative-only cohort reported an acute operative threshold or conversion-to-surgery criterion. Reports containing operative and nonoperative groups without a reported rationale for treatment assignment were retained and coded as having unreported selection criteria. Operative-only outcome series without treatment-selection information were excluded.

Both acute initial fixation decisions and conversion from an initial acute nonoperative strategy were eligible. Surgery for established nonunion, malunion, or chronic sequelae was excluded. Reviews, expert-opinion articles, surveys, biomechanical or cadaveric studies, single case reports, and conference abstracts without a full eligible report were excluded from extraction. English-language full text was required for final eligibility assessment.

**Analytic set versus background literature.** Surveys, biomechanical or cadaveric studies, commentaries, and previous reviews were excluded from the analytic set but could be cited as background literature; such citations contributed to no analytic denominator. Eligible reports could also be cited outside the Results for methodological or interpretive context. Such dual citation did not alter the study-level denominator, and companion reports remained linked within their corresponding study-level units.

### 2.3. Information Sources and Search Strategy

MEDLINE was searched via PubMed (National Library of Medicine, Bethesda, MD, USA) and Embase via Embase.com (Elsevier, Amsterdam, The Netherlands). The database searches used to construct the review dataset were executed on 12 August 2026 without date, language, age, species, publication-type, or study-design filters. Records were deduplicated electronically by matching DOI with a compatible title, or exact normalized title and publication year, and the result was cross-validated manually.

The final PubMed strategy was:

(“lateral compression”[tiab] OR LC1[tiab] OR “LC-1”[tiab] OR “LC-I”[tiab]) AND (pelvi*[tiab] OR sacral[tiab] OR sacrum[tiab])

The final Embase strategy was:

(‘lateral compression’:ti,ab,kw OR lc1:ti,ab,kw OR ‘lc 1’:ti,ab,kw OR ‘lc i’:ti,ab,kw) AND (pelvi*:ti,ab,kw OR sacral:ti,ab,kw OR sacrum:ti,ab,kw)

Search development included known-item validation, testing of a treatment-selection/instability block, incremental AO/OTA terminology testing, notation-variant testing, and an anatomy-block rescue analysis. A set of core validation records was used to test search sensitivity; sentinel status was used for search development only and did not confer eligibility, which was determined independently. Complete search histories and validation decisions are provided in the OSF search-strategy file.

Three supplementary methods were then applied. Reference lists of all 31 reports included at submission were screened; references were obtained from bibliographic export and, where deposited reference metadata were incomplete, directly from the published full text. Forward citation searching was performed with the Scopus (Elsevier, Amsterdam, The Netherlands) cited-by function for a prespecified set of six sentinel papers chosen to represent the principal operative-selection and instability-assessment approaches. Citation chasing was limited to one generation, and newly identified eligible reports were not used as additional citation seeds. Finally, both database searches were re-run unchanged on 14 August 2026. Records identified by these methods were first checked against the original screening set and against supplementary records already identified, and were screened only if new.

### 2.4. Screening Process and Verification

Screening was conducted in a single-human-reviewer workflow (H.K.S.) with generative-AI screening support, and the stages at which AI proposals were used were as follows. At title screening, ChatGPT (Section 2.7) received the bibliographic record of each of the 420 unique records together with the locked screening codebook and proposed either abstract review or exclusion, with a title-level code and a reason. Title-level proposals were not individually re-adjudicated. Instead, they were audited on 12 August 2026 (an AI-assisted audit reviewed by the human reviewer) by confirming that every prespecified sentinel record had been forwarded and by re-reviewing the 27 excluded titles that combined lateral-compression and treatment or instability terms; no erroneous exclusion was found. Of the 304 proposed title-level exclusions, 297 remained exclusions, four were reclassified as linkage-only registry or protocol records, and three were retained as background literature. At abstract screening, the model received the title, abstract, and bibliographic metadata of the 116 records forwarded from title screening and proposed *Include*, *Unclear*, or *Exclude*, with a detailed exclusion code, a reason, and the supporting abstract text. At the original screening, all 77 assistant-proposed abstract-level exclusions were reviewed and confirmed by the human reviewer, with no overrides; records proposed as *Include* or *Unclear* were advanced to full text, so that uncertainty was resolved in favor of full-text review. Full-text eligibility assessment, report linkage, and data extraction were performed by the human reviewer without AI proposals. Every AI proposal was recorded in dedicated columns of the screening workbook, separately from the human decision, and the workbook is deposited in the OSF audit archive; the model inputs and logging fields are described in Supplementary Methods S1. Records identified by the supplementary searches were screened in the same way. To check the screening, a second reviewer (J.L.), blinded to the original decisions, independently screened a random sample of 30 excluded records (20 excluded at title and 10 at abstract screening; fixed seed; drawn from the exclusions not included in an earlier packet that was withdrawn, see Supplementary Methods S1) at title-and-abstract level. The sample was interleaved with five records that had been advanced to full text, so that the proportion of exclusions could not be inferred; any excluded record that the second reviewer would have advanced was assessed at full text by the original reviewer. The second reviewer would have forwarded none of the 20 title-level exclusions, two of the 10 abstract-level exclusions, and all five advanced records. Both forwarded exclusions were judged eligible at full text by the original reviewer. Because both errors had arisen at abstract screening, the original reviewer then re-screened all 75 remaining abstract-level exclusions against the eligibility criteria, without AI proposals or access to the original decisions; eight were forwarded, of which four were eligible at full text. Full-text eligibility assessment and data extraction for these additional reports were again performed by the original reviewer without AI proposals.

**Figure 1:**
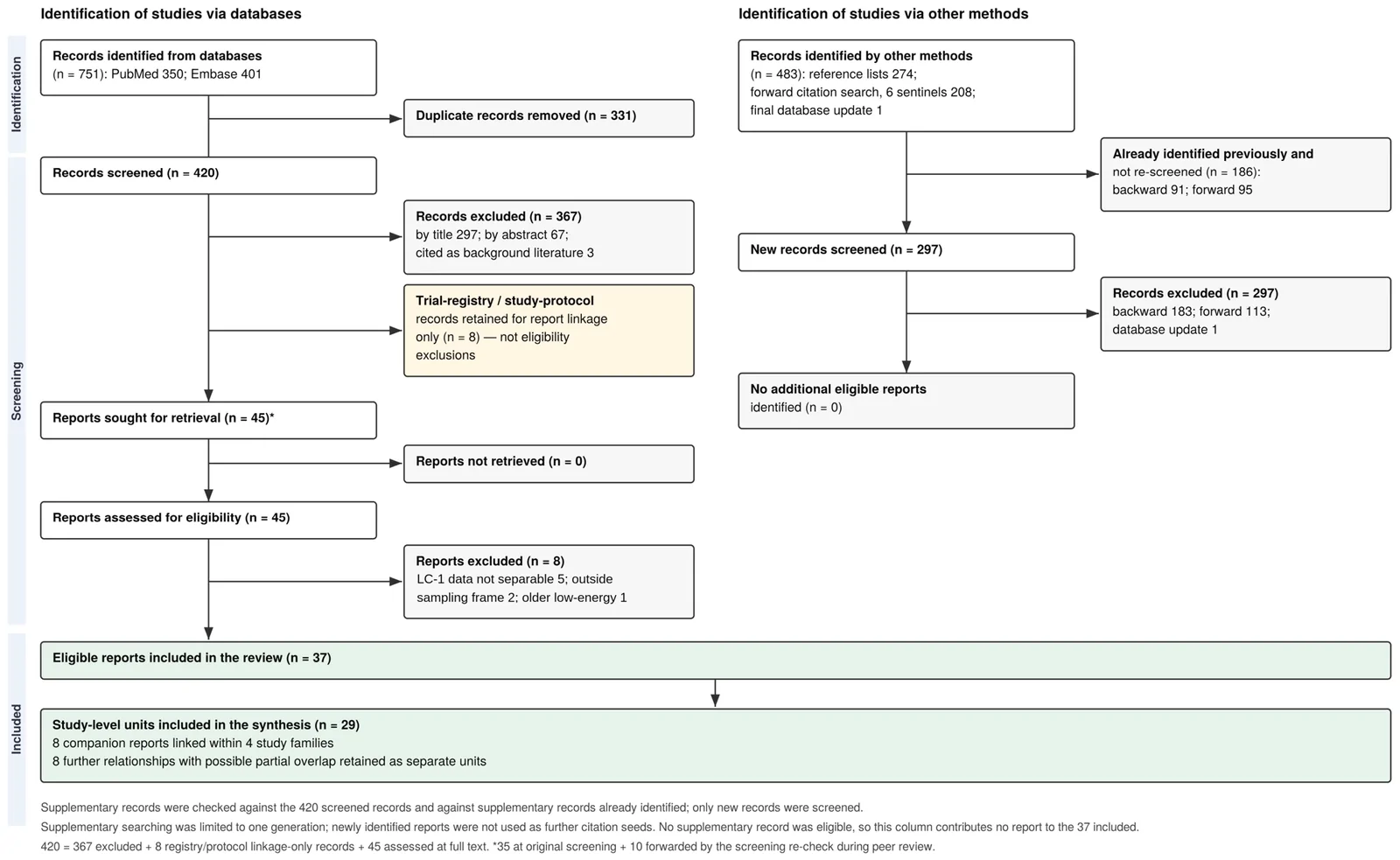
PRISMA 2020 flow diagram of study identification, screening, eligibility assessment, report linkage, and inclusion, including records identified by supplementary methods. Trial-registry and study-protocol records used for study linkage only (*n* = 8) are shown as a separate branch and were not treated as eligibility exclusions. Eight companion reports are linked within four study families, so that 37 eligible reports contribute 29 study-level units. Ten reports were assessed at full text after the screening re-check performed during peer review. The shaded yellow box denotes linkage-only records, and green boxes denote included reports and study-level units.

Trial-registry and study-protocol records were handled under the screening codebook as linkage-only records rather than eligibility-exclusion decisions and were not advanced as full-text reports; they were used only to link a protocol or registry entry to its parent study. Records reporting surveys, biomechanical work, or previous reviews that were excluded from the analytic set but cited for context were recorded separately as background literature. Full-text exclusion reasons were recorded and are reported for each excluded report in Table S2. Record counts at each stage are reported in Section 3.1 and Figure 1.

The initial screening and extraction decisions were subsequently subjected to second-pass verification and structured consistency checking. Generative AI systems were used only as assistance tools and were not treated as independent human reviewers; full-text eligibility, linkage, coding, and amendment decisions were made by the human author, and title-level exclusions were accepted subject to the audit described above.

### 2.5. Report Linkage and Unit of Analysis

The unit of analysis was the study-level unit, not the individual publication. Reports that described the same, reused, nested, or near-completely reused underlying cohort were linked and contributed a single study-level unit. Relevant information was combined across linked eligible reports, with source provenance retained in the Notes field.

Possible or probable partial participant overlap without sufficient evidence that the reports represented the same, reused, or nested analytic cohort was flagged but not merged. Because the eight-center multicenter series probably overlapped with selected earlier institutional series but the degree of participant overlap could not be quantified, a prespecified sensitivity analysis excluded that series from study-frequency summaries.

### 2.6. Data Extraction and Coding

One extraction row was created per study-level unit. The locked codebook contained 11 fields: study; design and comparative status; sample; LC-1 definition; operative indication; indication category; assessment method; quantitative threshold; selection-rule reconstructibility; notes; and population restriction relevant to displacement/instability.

Operative indications were classified as *Static*, *Dynamic*, *Clinical*, *Mixed*, *Unspecified*, *Discretionary*, *or Not reported*. Quantitative thresholds were recorded when explicitly linked to operative selection, recommendation, counseling, or consideration, even when the threshold did not automatically determine surgery. Population-only thresholds were recorded separately in the population-restriction field.

The prespecified codebook field termed *reporting explicitness* operationalized the reconstructibility of the operative-selection rule and is therefore referred to here as operative-selection rule reconstructibility, abbreviated to selection-rule reconstructibility; the coding categories and their prespecified definitions are unchanged. It was coded as *Explicit*, *Partial*, *or None*. Under a prespecified terminal-step rule, a treatment pathway ending in counseling, shared decision-making, or individualized judgment was classified as *Partial* because the operative-selection rule was not operationally reconstructible. For studies reporting multiple treatment pathways, all reported pathways had to have reconstructible triggers and treatment linkage for the study to be coded *Explicit*.

The discriminating feature was the terminal step of the reported pathway rather than the qualitative or quantitative form of the trigger. A qualitatively defined trigger whose reported pathway terminated in the operation actually performed was coded *Explicit*, whereas a fully quantified trigger whose pathway terminated in counseling, shared decision-making, or individualized judgment was coded *Partial*. Non-standardization of a criterion did not by itself lower the classification.

A *Partial* classification denotes that the assessment–rule–treatment sequence could not be fully reconstructed under the prespecified coding framework. It does not denote deficient reporting: a pathway may be reported in complete detail and still be non-deterministic, and under the terminal-step rule such a pathway is *Partial* by design.

Population restrictions were recorded only when eligibility or sampling directly restricted the study population by displacement, instability, a relevant morphologic or dynamic-stability feature, or the clinical construct used for operative selection (Table 1). Wording that merely restated the base LC-1/Tile diagnostic class without further narrowing the sampled population was retained in the injury-definition or Notes fields rather than coded as an additional population restriction. Because a population restriction and an operative threshold can carry the same numeric value while doing different work, the two are reported side by side in Table 2 so that a cohort entry criterion is not read as an operative decision rule.

### 2.7. Verification, Amendments, and Generative-AI Assistance

After the primary extraction, all 24 study-level units included at submission underwent structured re-verification against the locked codebook and the source reports. Verification included source re-reading of key treatment-selection passages, cross-study consistency checks, numerical checks, and report-linkage review. Targeted reconciliation covered selection-rule reconstructibility and population-restriction coding. All amendments were recorded in a dated audit trail rather than silently overwriting prior decisions; the complete amendment history is available in the OSF reconciliation record and extraction workbook.

Post-lock coding changes were documented in that dated amendment record. During structured verification, the primary outcome classification changed in 2 of 24 study-level units; both changes applied the prespecified terminal-step rule. This record documents coding verification within the review process and is distinct from the between-reviewer reproducibility assessment described below.

The prespecified review process did not include duplicate independent coding or reliability estimation. To evaluate the reproducibility of the final coding framework, we subsequently conducted a post hoc independent recoding of the 24 study-level units included at submission under a separately fixed analysis plan. A second orthopaedic surgeon (J.L.), based at a different institution and having taken no part in the screening, extraction, coding, or drafting that preceded this assessment, recoded indication category, quantitative threshold, and selection-rule reconstructibility from the locked codebook and the source reports, blinded to the original coding, the locked dataset, the amendment record, the analysis outputs, and the manuscript. After a calibration exercise on four reports excluded at full text, no study-specific discussion or feedback was permitted, and the second coder’s entries were fixed before any comparison was made. The second coder joined the study as an author only after the recoding had been submitted and fixed. The five units added during peer review (Section 2.9) were recoded by the same coder under the same blinded procedure, without further calibration, by which time he was a co-author; his entries were again fixed before any comparison was made. This assessment is not equivalent to prospective duplicate review; its procedural details are given in Supplementary Methods S1.

Disagreements were classified by the original reviewer (H.K.S.) after the second coder’s entries had been fixed, using two types defined in the reproducibility analysis plan before any comparison was made: Type A, a clear misapplication of the codebook or an error of source extraction, which would be corrected; and Type B, an interpretive disagreement possible within the locked coding rules, for which the original coding would be retained, and the disagreement reported. No consensus meeting was held, and the second coder took no part in this classification. Agreement was calculated on the codes as they stood before classification and is therefore unaffected by it.

Two generative-AI systems were used in separate supporting roles. ChatGPT (OpenAI, San Francisco, CA, USA; model/version: GPT-5.6 Sol) was used for structured screening support, extraction-coding cross-checks, workbook and data reconciliation, consistency checking, audit trail generation, and language drafting. Claude (Anthropic, San Francisco, CA, USA; model/version: Claude Opus 5) was used for a separate AI-assisted full-text and source-verification pass across the final study set and for workbook and package integrity auditing; this process generated discrepancy flags that prompted human re-checking of source papers and project records. Neither system was treated as an independent human reviewer or permitted to make final coding or full-text eligibility decisions; at title screening, assistant-proposed exclusions were accepted subject to the audit described in Section 2.4 rather than individually re-adjudicated, and at abstract screening each proposed exclusion was reviewed by the human reviewer. All source interpretation, adjudication, amendment approval, and final manuscript content were reviewed and approved by the human authors.

### 2.8. Data Synthesis

Synthesis was descriptive and used study-level units as the denominator. Counts and proportions were calculated for indication category and selection-rule reconstructibility, and assessment methods and quantitative thresholds were summarized narratively because multiple methods or thresholds could occur within a single study. Assessment method proportions are therefore non-mutually exclusive and sum to more than 100%.

No meta-analysis, treatment-effect estimate, statistical heterogeneity measure, formal certainty-of-evidence assessment, or formal risk-of-bias tool was applied. These procedures were not considered aligned with the review objective, which was to map reported operative-selection criteria and their operational definitions rather than estimate treatment effects. Selection-rule reconstructibility was treated as a review outcome and deliberately not used as a risk-of-bias or quality score, since using the review’s own outcome as a quality metric would render the analysis circular.

The final dataset comprised 29 study-level units representing 37 eligible reports (24 units and 31 reports at submission; Section 2.9). A sensitivity analysis repeated study-frequency summaries after excluding the eight-center multicenter series because of probable but unquantifiable participant overlap with earlier institutional series.

Two further analyses were added post hoc after completion of the prespecified synthesis and are labeled as such throughout. First, units not classified as *Explicit* were decomposed into the three components of the *Explicit* definition—whether the assessment or candidate variable, the rule defining a positive finding, and the linkage from that finding to operative treatment could each be reconstructed. The components are non-exclusive: a unit may fail at more than one. Second, two post hoc sensitivity analyses were performed: one collapsing the four units drawn from a single institutional lateral-stress-radiograph registry into one cluster, and one restricted to units in which a clinical operative-selection pathway was applicable by design. In the cluster analysis, the umbrella registry report was retained as the single representative of that registry and the three further reports from it were dropped, so that repeated reporting from one institutional source contributed one occurrence rather than four; no unit outside that registry was affected.

For the reproducibility assessment, agreement was calculated on pre-adjudication codes as percent agreement with Wilson 95% confidence intervals and, secondarily, as unweighted Cohen’s κ with bootstrap 95% confidence intervals obtained by resampling study-level units (resampling, software, and seed are specified in Supplementary Methods S1). Statistical analyses were performed using SPSS (version 29.0; IBM Corp., Armonk, NY, USA), and the bootstrap confidence intervals for κ were computed in Python (version 3.11; Python Software Foundation, Wilmington, DE, USA). Because the original coding was highly imbalanced across categories and the sample comprised only 29 units, κ was expected to be sensitive to category prevalence and to individual disagreements; percent agreement is therefore the primary descriptive measure and κ is interpreted with caution. As a sensitivity analysis prespecified within this post hoc assessment, agreement was recalculated in the units remaining after exclusion of five units potentially exposed by the codebook’s worked examples: the eight-center multicenter series [17], the emergency-department stress cohort of DeKeyser et al. [3,18], and the three units whose primary report is a 2023 publication from the Denver lateral-stress-radiograph group (the departmental stress-protocol, lateral-stress-radiograph registry, and stress-positive LC1b cohorts in Table 1). The worked examples name the first two directly but refer to the 2023 Denver report without indicating which of these three units is meant, so all three were excluded conservatively. Results under the second coder’s codes are reported alongside the primary results (Section 3.8, Table 3).

**Table 1:** Characteristics of the 29 included study-level units.

Study	Contributing Eligible Reports [Ref]	Design	LC-1 Cohort	LC-1 Definition	Population Restriction Relevant to Instability/Displacement
Sagi et al. (USA)	[1]	Retrospective cohort; non-comparative	*n* = 19 (7 operative/12 nonoperative); mean age 35 y overall	Young–Burgess LC1/OTA 61-B2; sacral impaction/incomplete posterior-ring injury	Presumed-stable nondisplaced sacral alar impaction was not taken for EUA
Orthopaedic Trauma Research Group (multicenter)	[17]	Multicenter retrospective comparative cohort	*n* = 224 (139/85); median age 47 vs. 59 y	Minimally displaced stress-positive LC1; OTA/AO 61B2; <1 cm rami displacement	Initial rami displacement < 1 cm; stress-positive injuries only
DeKeyser et al. (USA)	[3,18]	Prospective cohort + linked retrospective reanalysis; non-comparative	*n* = 70 (15/55); mean age 59.2 y; low-energy 46%	Author-defined minimally displaced LC1; <10 mm displacement	Initial displacement < 10 mm
Tucker et al. (USA; departmental stress protocol)	[19]	Retrospective pre/post-protocol comparative cohort	*n* = 73 (18/55); median age 56 y pre-protocol vs. 47 y LSR	Acute isolated minimally displaced LC1; <10 mm static displacement	Static displacement < 10 mm
Tucker et al. (USA; Denver LSR registry)	[2,20,21,22,23,24]	Retrospective registry cohort; non-comparative	*n* = 128 (53/75); median age 57 y; GLF 23.4%	Minimally displaced LC1; <10 mm static displacement	Primary report: static displacement < 10 mm
Patterson et al. (USA)	[4]	Prospective two-center diagnostic trial; comparative secondary analysis	*n* = 31 (10/21); mean age 58.7 y; GLF 32.3%	OTA/AO B1.1/2.1 LC1 confirmed by X-ray/CT	None stated
Tucker et al. (USA; comminuted-rami cohort)	[25]	Retrospective registry cohort; comparative	*n* = 125 (49/76); median age 50 y; high-energy 58.4%	Minimally displaced LC1; <1 cm static displacement	Static displacement < 1 cm
Hoskins et al. (USA)	[26]	Retrospective two-center operative cohort; non-comparative	Analytic *n* = 120, all operative; source LC-1 cohort *n* = 333	Young–Burgess LC1; author-mapped to AO/OTA 61-B1/B2	Operative-only cohort selected for radiographic instability or failed mobilization
Ellis et al. (USA)	[27]	Retrospective cohort; comparative	*n* = 161 (86/75); LC-1-specific age NR	Young–Burgess LC1 within traumatic Tile B/OTA-AO 61B LC cohort	None stated
Rusu et al. (USA)	[28]	Retrospective administrative cohort; comparative	*n* = 72,767 (7324/65,443)	ICD-9/10 code algorithm for closed LC1 pattern; AO/OTA 61B2.1 referenced	None stated
Slobogean et al. (USA)	[29,30]	Prospective trial with randomized and preference arms; comparative	LC-1 *n* = 67 (31/36); overall trial mean age 43.5 y	Young–Burgess LC; OTA/AO 61-B1/B2; complete posterior-ring fracture < 10 mm	Complete posterior-ring fracture; initial displacement < 10 mm
Höch et al. (Germany)	[31]	Retrospective comparative cohort	*n* = 71 (36/35); mean age 37.3 y; high-energy 93%	AO/Tile B2-1, described as Young–Burgess LC1	None stated
Tucker et al. (USA; stress-positive LC1b cohort)	[32]	Retrospective registry cohort; comparative	*n* = 43 (27/16); median age 62 y; low-energy 55.8%	LC1b: <10 mm static + ≥10 mm dynamic displacement on LSR	Static displacement < 10 mm and dynamic displacement ≥ 10 mm
Metzger et al. (Germany)	[33]	Single-center observational cohort; comparative	*n* = 77 (39/38); median age 54 vs. 57 y	Traumatic Young–Burgess LC1/AO/OTA 61B1.1	None stated
Sembler Soles et al. (USA)	[34]	Retrospective nonoperative cohort; non-comparative	*n* = 118 initially nonoperative; 1 converted; mean age 46 y	Young–Burgess LC1; <10 mm initial displacement	Initial displacement < 10 mm
Therrien et al. (Netherlands)	[35]	Prospective cohort; comparative	*n* = 203 (24/179); high-energy 64%; overall age NR	Young–Burgess LC1; CT injury-severity score 5–14	None stated
Whiting et al. (USA)	[36]	Retrospective two-center diagnostic cohort; non-comparative	Negative-EUA cohort: LC-1 *n* = 22 at screening; analytic LC-1 *n* = 16	Young–Burgess LC1 with complete sacral fracture	Complete sacral fracture; negative EUA
Tosounidis et al. (UK)	[37]	Prospective cohort; comparative	*n* = 63 (35/28); median age 31.4 vs. 37.6 y	Young–Burgess LC1; OTA 61-B1	None stated
Hagen et al. (USA; with companion report)	[38,39]	Retrospective matched cohort; comparative	*n* = 158 (51/107); mean age 35 vs. 41 y	Young–Burgess LC1 defined by sacral impaction fracture	Nonoperative comparator: complete sacral fracture; companion subset < 1 cm initial displacement
Zhang et al. (China)	[5]	Prospective cohort; comparative	*n* = 22 (9/13); mean age 53.1 y	Tile-based LC1 diagnostic criteria; described as partially stable LC fracture	None stated
Shawky et al. (Egypt)	[40]	Prospective case series; non-comparative	*n* = 25 (22 unstable-operative/3 stable-nonoperative)	Young–Burgess LC1 with complete sacral fracture	Complete sacral fracture; incomplete sacral and vertically unstable fractures excluded
Huang et al. (China)	[41]	Historical-control study; comparative	*n* = 77 (44/33); mean age 53.3 y	Tile-based LC1 criteria; described as partially stable LC1	None stated
Brodell et al. (USA)	[42]	Retrospective cohort; comparative	LC-1 *n* = 438; LC-1 operative/nonoperative counts NR	Young–Burgess LC I	None stated
Burgess et al. (USA)	[7]	Consecutive protocol-based observational cohort; non-comparative	LC-I *n* = 79; mean age 31.1 y; high-energy cohort	Young LC-I: anterior transverse ramus fracture + ipsilateral sacral compression	Sampling required interruption of both anterior and posterior pelvic ring
Abdelrahman et al. (Germany)	[43]	Retrospective single-center operative cohort; non-comparative	Analytic *n* = 37, all operative (23 posterior-only/14 combined); source LC-1 cohort reported as *n* = 226; mean age 70 y	Young–Burgess LC1 confirmed on CT	Operatively treated LC-1; standardized percutaneous fixation only
Brostowitz et al. (USA)	[44]	Retrospective registry cohort; comparative	*n* = 156 (15/141); mean age 58.7 y; ground-level fall 32.0%	LC-1 as classified in the institutional trauma registry	None stated
Beckmann et al. (USA)	[45]	Survey-derived radiographic score with patient validation cohort; comparative	Validation cohort *n* = 33; derivation: 27 LC-1 cases rated by 111 surgeons	LC-1; score based on sacral and ramus fracture morphology	None stated
Lawson et al. (USA)	[46]	Retrospective nonoperative series; non-comparative	*n* = 173 nonoperative (139 with complete outpatient radiographs); mean age 50 y; ground-level fall 15.0%	OTA/AO 61-B1 LC1	Stable injuries selected for nonoperative care with stable postmobilization radiographs
Avilucea et al. (USA; multicenter)	[47]	Multicenter retrospective cohort; predefined algorithm applied prospectively; non-comparative	LC-1 *n* = 53, all operative (28 combined/25 posterior-only); mean age 41 y overall	Young–Burgess LC1	Unstable on EUA (>1 cm); EUA-stable injuries excluded

Abbreviations: AO/OTA, AO Foundation/Orthopaedic Trauma Association; CT, computed tomography; EUA, examination under anesthesia; GLF, ground-level fall; LC-1, lateral compression type 1; LSR, lateral stress radiograph; NR, not reported. Note 1: Study-level units combine linked eligible reports when they represent the same, reused, nested, or near-completely reused underlying cohort. Note 2: Population restrictions are reported only when they limited the sampled population by displacement, instability, morphology, or the operative-selection clinical construct. Note 3: For study-level units formed from linked reports, all eligible reports contributing to that unit are cited in the “Contributing eligible reports” column. Eight companion reports are linked within four units; the analytic denominator is 29 study-level units.

**Table 2:** Reported operative-selection criteria, assessment methods, population restrictions, quantitative thresholds, and selection-rule reconstructibility for LC-1 pelvic ring injuries (29 study-level units).

Study	[Ref]	Operative-Selection Criterion/ Pathway	Category	Assessment Method	Population Restriction Relevant to Instability/ Displacement	Threshold	Selection-Rule Reconstructibility (Prespecified “Reporting Explicitness” Category)
Sagi et al. (USA)	[1]	EUA overlap > 1 to <2 cm → anterior fixation; >2 cm → anterior + posterior fixation.	*Dynamic*	EUA/fluoroscopy	Presumed-stable nondisplaced sacral alar impaction was not taken for EUA	>1 cm for any fixation; >2 cm for anterior + posterior fixation	*Explicit*
Orthopaedic Trauma Research Group (multicenter)	[17]	Stress-positive ED/LSR → counseling/shared decision; positive EUA → routine pelvic fixation.	*Mixed*	ED stress/LSR/EUA	Initial rami displacement < 1 cm; stress-positive injuries only	NR; stress-positive threshold varied across centers	*Partial*
DeKeyser et al. (USA)	[3]	ED stress ≥ 10 mm → fixation; stress-negative patients with intolerable pain/inability to walk could undergo posterior fixation.	*Mixed*	ED stress + EUA + mobilization	Initial displacement < 10 mm	ED stress ≥ 10 mm; clinical conversion trigger qualitative	*Explicit*
Tucker et al. (USA; departmental stress protocol)	[19]	After LSR implementation, dynamic displacement ≥ 1 cm → routine operative management.	*Dynamic*	LSR; occasional EUA	Static displacement < 10 mm	LSR ≥ 1 cm	*Partial*
Tucker et al. (USA; Denver LSR registry)	[20]	Static displacement ≥ 10 mm → fixation without EUA (departmental protocol reported in companion report [24]). LSR ≥ 10 mm → operative counseling/consideration; <10 mm → nonoperative unless clinical failure. Temporary mobilization protocol: unable to walk 15 ft by day 2 → surgery.	*Mixed*	Static X-ray + LSR + mobilization	Primary report: static displacement < 10 mm	Static ≥ 10 mm; LSR ≥ 10 mm; inability to walk 15 ft by day 2 in temporary protocol	*Partial*
Patterson et al. (USA)	[4]	Treatment remained surgeon-discretionary; PBR was diagnostic rather than a prespecified allocation rule, with treatment modified by pain/mobilization and patient choice.	*Mixed*	Pelvic-binder stress radiography + mobilization	None stated	NR; ≥1 cm at 10 kg was diagnostic/reference, not an allocation rule	*Partial*
Tucker et al. (USA; comminuted-rami cohort)	[25]	LSR dynamic displacement ≥ 1 cm → operative counseling; <1 cm → nonoperative management.	*Dynamic*	LSR	Static displacement < 1 cm	LSR ≥ 1 cm	*Partial*
Hoskins et al. (USA)	[26]	Radiographic instability or inability to ambulate 100 ft within 3 days → surgery offered.	*Mixed*	X-ray/CT + mobilization	Operative-only cohort selected for radiographic instability or failed mobilization	Unable to walk 100 ft within 3 days; radiographic threshold NR	*Partial*
Ellis et al. (USA)	[27]	Treatment by surgeon preference; positive EUA supported surgery when performed, but EUA selection and other pathways were not standardized.	*Mixed*	EUA/fluoroscopy + surgeon judgment	None stated	NA; qualitative instability/displacement	*Partial*
Rusu et al. (USA)	[28]	Operative/nonoperative status identified by codes; treatment-selection basis not reported.	*Not reported*	NR	None stated	NR	*None*
Slobogean et al. (USA)	[29]	Allocation by randomization when accepted; otherwise patient preference under clinical equipoise, without a clinical operative indication.	*Discretionary*	NA	Complete posterior-ring fracture; initial displacement < 10 mm	NA	*None*
Höch et al. (Germany)	[31]	Greater anterior-ring displacement was associated with surgery, but no reproducible patient-level cutoff or treatment rule was reported.	*Static*	CT	None stated	NR	*Partial*
Tucker et al. (USA; stress-positive LC1b cohort)	[32]	LSR ≥ 10 mm → counseling on surgery; fixation performed if the patient elected surgery.	*Mixed*	LSR + shared decision	Static displacement < 10 mm and dynamic displacement ≥ 10 mm	LSR ≥ 10 mm	*Partial*
Metzger et al. (Germany)	[33]	Surgeon discretion based on morphology, radiographic instability, and clinical/mobilization status; conversion for failed mobilization or secondary displacement.	*Mixed*	X-ray/CT + mobilization/clinical assessment	None stated	NA	*Partial*
Sembler Soles et al. (USA)	[34]	Conversion considered for interval displacement accompanied by substantial pain and failure to mobilize.	*Mixed*	Serial X-ray + mobilization/pain	Initial displacement < 10 mm	NA; qualitative displacement + pain/mobilization failure	*Partial*
Therrien et al. (Netherlands)	[35]	Standard-practice decision based on fracture pattern, pain, mobilization, and patient preference; CT score was not used for treatment selection.	*Mixed*	X-ray/CT + mobilization/clinical assessment	None stated	NA	*Partial*
Whiting et al. (USA)	[36]	EUA overlap > 1 cm → operative criteria met; negative EUA → treatment without internal fixation.	*Dynamic*	EUA/fluoroscopy	Complete sacral fracture; negative EUA	EUA > 1 cm overlap	*Explicit*
Tosounidis et al. (UK)	[37]	MUA/EUA translation > 2 cm → operative treatment; lesser displacement → nonoperative treatment.	*Dynamic*	MUA/EUA/fluoroscopy	None stated	MUA/EUA > 2 cm translation	*Explicit*
Hagen et al. (USA; with companion report)	[38]	Generally >10 mm initial displacement and/or sagittal/axial deformity → surgery; final decision remained discretionary ± patient/family input.	*Mixed*	X-ray/CT + clinical/associated-injury context	Nonoperative comparator: complete sacral fracture; companion subset < 1 cm initial displacement	>10 mm initial displacement (general threshold)	*Partial*
Zhang et al. (China)	[5]	Individualized decision using mechanism, classification, pain, displacement, patient demand, and ultrasound stability; mobility ≥ 0.3 cm defined instability and informed operative consideration.	*Mixed*	Ultrasound stress + X-ray/CT + clinical assessment	None stated	Ultrasound mobility ≥ 0.3 cm	*Partial*
Shawky et al. (Egypt)	[40]	Sequential EUA: stress displacement ≥ 1 cm = unstable → posterior fixation; add anterior fixation if instability persisted.	*Dynamic*	Sequential EUA/fluoroscopy	Complete sacral fracture; incomplete sacral and vertically unstable fractures excluded	EUA ≥ 1 cm stress displacement	*Explicit*
Huang et al. (China)	[41]	TES: multifactorial discretionary selection. CUE: ultrasound mobility ≥ 0.3 cm defined instability, but final treatment remained surgeon-selected.	*Mixed*	X-ray/CT + ultrasound stress + clinical assessment	None stated	Ultrasound mobility ≥ 0.3 cm; TES threshold NR	*Partial*
Brodell et al. (USA)	[42]	LC-I injuries considered at increased instability underwent EUA; 1 cm stress displacement = positive test → fixation if indicated.	*Dynamic*	EUA	None stated	1 cm stress displacement	*Partial*
Burgess et al. (USA)	[7]	LC-I was generally nonoperative; external fixation was reserved for prolonged hemodynamic instability or associated injuries benefiting from early mobilization.	*Clinical*	Clinical/hemodynamic assessment	Sampling required interruption of both anterior and posterior pelvic ring	NA; qualitative clinical triggers	*Partial*
Abdelrahman et al. (Germany)	[43]	Incomplete sacral fracture → supervised mobilization; nonoperative if weight bearing tolerated. Posterior fixation if pain prevents mobilization or CT shows a complete sacral fracture; anterior fixation added for predominant anterior pain, particularly with comminution, an oblique ramus fracture line, or static ramus displacement > 5 mm.	*Mixed*	CT + mobilization trial + clinical assessment	Operatively treated LC-1; standardized percutaneous fixation only	Static ramus displacement > 5 mm (anterior fixation)	*Partial*
Brostowitz et al. (USA)	[44]	Surgical or nonsurgical treatment planned at the attending surgeon’s discretion; no selection criterion reported.	*Discretionary*	NA	None stated	NR	*None*
Beckmann et al. (USA)	[45]	Radiographic LC-1 score: <7 → nonoperative recommendation; >9 → operative recommendation; 7–9 indeterminate, requiring further evaluation.	*Static*	X-ray/CT morphology score	None stated	Score < 7/ > 9	*Partial*
Lawson et al. (USA)	[46]	Minimally displaced ( < 1 cm) → in-hospital mobilization; displaced → fixation; instability on postmobilization radiographs not defined quantitatively.	*Mixed*	X-ray + mobilization and postmobilization radiographs	Stable injuries selected for nonoperative care with stable postmobilization radiographs	Static displacement < 1 cm (mobilization pathway)	*Partial*
Avilucea et al. (USA; multicenter)	[47]	Initial EUA > 1 cm ramus overlap → posterior fixation; repeat EUA with >1 cm residual displacement → anterior fixation.	*Dynamic*	Sequential EUA/fluoroscopy	Unstable on EUA (>1 cm); EUA-stable injuries excluded	EUA > 1 cm	*Explicit*

Abbreviations: CT, computed tomography; ED, emergency department; EUA, examination under anesthesia; LSR, lateral stress radiograph; MUA, manipulation under anesthesia; NA, not applicable; NR, not reported; PBR, pelvic-binder radiography; TES, traditional experience strategy; CUE, combined ultrasonography examination. Note 1: A quantitative threshold linked to counseling or operative consideration is retained in its original decision context and should not be interpreted as a universal operative mandate. Note 2: *Explicit* = assessment/variable + positive rule + actual operative linkage reconstructible; *Partial* = one or more elements incomplete; *None* = no operationally usable operative decision rule reconstructible. Note 3: *Mixed* indicates ≥2 distinct selection mechanisms/pathways within the study; assessment method counts in the Results are therefore non-mutually exclusive. Note 4: Population restriction is reported immediately before the threshold because the two are distinct: a restriction defines who entered the cohort, whereas a threshold defines what triggered operative selection within it. Reading a threshold such as “LSR ≥ 10 mm” without the accompanying restriction “static displacement < 10 mm” misattributes the cohort entry criterion to the operative rule. Note 5: Citation numbers correspond to the primary report of each study-level unit; the complete set of eligible reports contributing to each unit is cited in Table 1.

**Table 3:** Principal results under the original coding and under the second coder’s independent coding (29 study-level units).

Result	Original Coding	Second Coder’s Coding
Indication category	*Mixed* 15 (51.7%); *Dynamic* 8 (27.6%); *Static* 2 (6.9%); *Discretionary* 2 (6.9%); *Clinical* 1 (3.4%); *Not reported* 1 (3.4%); *Unspecified* 0	*Mixed* 17 (58.6%); *Dynamic* 7 (24.1%); *Not reported* 3 (10.3%); *Static* 1 (3.4%); *Discretionary* 1 (3.4%); *Clinical* and *Unspecified* 0
Quantitative threshold reported	18 (62.1%)	19 (65.5%)
Dynamic threshold of approximately 1 cm	10 (34.5%)	11 (37.9%)
Selection-rule reconstructibility	*Explicit* 6 (20.7%); *Partial* 20 (69.0%); *None* 3 (10.3%)	*Explicit* 7 (24.1%); *Partial* 17 (58.6%); *None* 5 (17.2%) ^a^
Non-reconstructible component among *Partial* units ^b^	*n* = 20: assessment 1; positive rule 13; linkage 15; positive rule or linkage 20/20	*n* = 17: assessment 0; positive rule 11; linkage 13; positive rule or linkage 17/17

^a^ Two of the five units classified as *None* by the second coder reflect designs in which no clinical operative-selection pathway could be ascertained (an administrative claims analysis and a trial allocating by randomization or patient preference). ^b^ The second coder recoded the three prespecified fields but not the post hoc component decomposition; the original component coding was therefore applied to the units the second coder classified as *Partial*. The 21 units with an applicable but incompletely reconstructible pathway described in Section 3.6 comprise the 20 units classified as *Partial* under the original coding and one unit classified as *None* in which treatment was left to surgeon discretion.

### 2.9. Deviations from the Review Plan

The reference-list screening, forward citation searching, and database update prespecified in the review plan and described in Section 2.3 were completed after the primary synthesis had been assembled rather than before it. They identified no additional eligible report.

During peer review, the blinded sample check of screening exclusions described in Section 2.4 identified two eligible reports that had been excluded at abstract screening, and the resulting re-screening of all abstract-level exclusions identified four more. These six reports were added under the locked eligibility criteria and codebook, without change to either: five formed new study-level units and one was linked as a companion report to an existing registry unit, so that the review comprises 37 eligible reports and 29 study-level units. The companion report described a further pathway in that registry’s protocol (static displacement of 10 mm or more leading to fixation), which was added to the unit’s operative-selection, assessment method, and threshold entries; its indication category and selection-rule reconstructibility were unchanged, and no coded value of any other original unit was changed. Title-level exclusions were not re-screened, because none of the 20 sampled title-level exclusions was disputed. The supplementary citation searches were not repeated for the six added reports. The five new units were coded by the original reviewer and independently recoded by the second coder under the same blinded procedure (Section 2.7). All analyses reported here use the final 29 units.

The component decomposition of non-*Explicit* units, the two sensitivity analyses described in Section 2.8, and the reproducibility assessment described in Section 2.7 were added after completion of the prespecified synthesis, to further characterize the findings and to assess the robustness and reproducibility of the final coding framework, and are identified as post hoc wherever they are reported. These post hoc analyses changed no eligibility criterion, coding rule, unit of analysis, or coded value.

**Timeline.** Because registration was retrospective, the dated sequence of the review is reported here; the full dated record is given in Supplementary Methods S1. The review plan and codebook were locked, the database searches executed, and title and abstract screening performed on 12 August 2026. Full-text assessment, data extraction, and the initial data lock followed on 13 August 2026, and structured re-verification, post-lock amendments, and the final data lock on 13–14 August 2026. The OSF registration and the supplementary searches were completed on 14 August 2026. On 18 August 2026, the human confirmation of the 77 assistant-proposed abstract-level exclusions was formally recorded in the screening workbook, the second coder’s independent recoding was returned and fixed, and the disagreements were classified; the manuscript was submitted the same day. During peer review, in October 2026, the blinded sample check of screening exclusions, the re-screening of abstract-level exclusions, full-text assessment and extraction of the additional reports, and the second coder’s recoding of the five new units were completed; the dated record is given in Supplementary Methods S1.

## 3. Results

### 3.1. Study Selection

The database searches identified 751 records, including 350 from PubMed and 401 from Embase. After removal of 331 duplicates, 420 unique records underwent title and abstract screening. A total of 367 records were excluded during these stages (297 at title and 67 at abstract screening, and three retained as background literature and cited as such rather than analyzed). A further eight trial-registry or study-protocol records were retained solely for report linkage and were not treated as eligibility exclusions. Forty-five reports underwent full-text assessment—35 at the original screening and 10 forwarded by the screening re-check during peer review (Section 2.4). Eight were excluded: five because LC-1-specific treatment-selection information could not be separately extracted from broader or mixed lateral-compression populations, two because they fell outside the treatment-selection sampling frame, and one because the cohort was restricted to low-energy injuries in older adults; each excluded report is listed with its citation and reason in Table S2. Accordingly, 37 reports met the eligibility criteria (Figure 1).

Nineteen of the 420 unique records were non-English publications, identified by the translated-title notation of their bibliographic records. Thirteen were excluded at title screening on content grounds, and six at abstract screening—five because only non-English full text was available and one on population grounds. No non-English report reached full-text assessment.

Supplementary methods added 297 further records for screening—183 from reference-list checking of the 31 reports included at submission, 113 from forward citation searching of six sentinel papers, and one from the final database update. All 297 were excluded, and no additional eligible report was identified.

Eight secondary or companion reports were linked within four study families because they represented the same, reused, nested, or near-completely reused underlying cohorts. The 37 eligible reports therefore contributed 29 study-level units to the primary synthesis. Eight additional relationships with possible or partial participant overlap were documented but retained as separate study-level units because the available evidence did not establish that they represented the same analytic cohort.

### 3.2. Study Characteristics

The 29 study-level units were published between 1990 and 2026 (Table 1). Twenty studies (69.0%) were conducted in the United States, three (10.3%) in Germany, two (6.9%) in China, and one each (3.4%) in the Netherlands, the United Kingdom, and Egypt. The remaining study (3.4%) was a multicenter series conducted across eight Level I trauma centers for which site-level national attribution was not specified in the report. Eighteen studies (62.1%) included a formal operative-versus-nonoperative or conservative comparison, whereas eleven (37.9%) were non-comparative.

The included studies were methodologically heterogeneous and comprised prospective and retrospective institutional cohorts, stress-imaging and diagnostic studies, protocol-based observational cohorts, a prospective clinical trial, and a large administrative database study. Definitions of LC-1 also varied, including Young–Burgess, AO/OTA or Tile-based classifications, and author-defined minimally displaced or morphology-based criteria. Several studies further restricted inclusion according to baseline displacement, sacral fracture morphology, dynamic instability, or other stability-related features.

### 3.3. Reported Operative-Selection Criteria

Operative-selection pathways were predominantly multifactorial (Table 2). Fifteen of 29 study-level units (51.7%) were classified as *Mixed*, indicating the use of two or more distinct selection mechanisms or decision pathways. Eight units (27.6%) were classified as *Dynamic*. *Static* and *Discretionary* indications each accounted for two units (6.9%) and *Clinical* for one (3.4%). In one unit (3.4%), the basis for operative selection was not reported. Although *Unspecified* was retained as a prespecified category, no unit was ultimately assigned to it.

Mixed pathways incorporated varying combinations of fracture morphology or displacement, dynamic stress testing, pain, mobilization failure, associated injuries, surgeon judgment, patient preference, and shared decision-making. Thus, the presence of an objective measure of instability did not necessarily translate into deterministic operative allocation.

### 3.4. Assessment Methods

Assessment methods were non-mutually exclusive because individual studies could use more than one method within the same treatment-selection pathway; percentages are calculated using the 29 study-level units as the denominator and therefore sum to more than 100%. Ten studies (34.5%) used examination or manipulation under anesthesia and/or dynamic fluoroscopy. Seven (24.1%) used awake stress radiography, including emergency-department stress radiographs, lateral stress radiographs, or pelvic-binder radiography. Static radiographic or CT morphology/displacement contributed to treatment selection in twelve studies (41.4%), while mobilization or functional clinical assessment was used in nine (31.0%). Two studies (6.9%) used ultrasonography with pelvic compression/separation stress testing, and one historical study (3.4%) relied primarily on clinical or hemodynamic circumstances.

Overall, 16 of 29 study-level units (55.2%) incorporated some form of dynamic or stress assessment when examination/manipulation under anesthesia, awake stress radiography, and stress ultrasonography were considered together.

### 3.5. Quantitative Thresholds

A quantitative threshold explicitly linked to operative selection, operative recommendation, counseling, or consideration was reported in 18 of 29 study-level units (62.1%). Six studies (20.7%) used qualitative criteria for which a numeric threshold was not applicable, and five (17.2%) did not report a usable quantitative threshold.

Dynamic displacement of approximately 1 cm (10 mm) was the most recurrent quantitative criterion, appearing in ten of 29 study-level units (34.5%). In a conservative post hoc sensitivity analysis collapsing four units from a single institutional registry into one cluster, this was reduced to seven cluster-level occurrences. This threshold was used across several assessment settings, including emergency-department stress radiography, lateral stress radiography, and examination under anesthesia. Its clinical meaning, however, varied substantially. In some studies, exceeding approximately 1 cm was directly linked to operative fixation, whereas in others it triggered counseling, operative consideration, or shared decision-making rather than mandatory surgery.

The same numerical value also served two distinct measurement constructs: approximately 10 mm was applied to dynamic stress displacement in the ten units above, and to static initial displacement as a general operative threshold or pathway boundary in three units, one of which applied both constructs in sequence.

Other reported quantitative criteria included >2 cm of displacement during manipulation or examination under anesthesia, ultrasonographic left-right mobility of ≥0.3 cm, static pubic-ramus displacement > 5 mm for supplementary anterior fixation, cutoffs on a radiographic morphology score, and mobilization-based thresholds such as failure to ambulate a specified distance within an early post-injury period. These findings demonstrate that even when numerical criteria were provided, their operational role differed across studies.

### 3.6. Selection-Rule Reconstructibility

**Figure 2:**
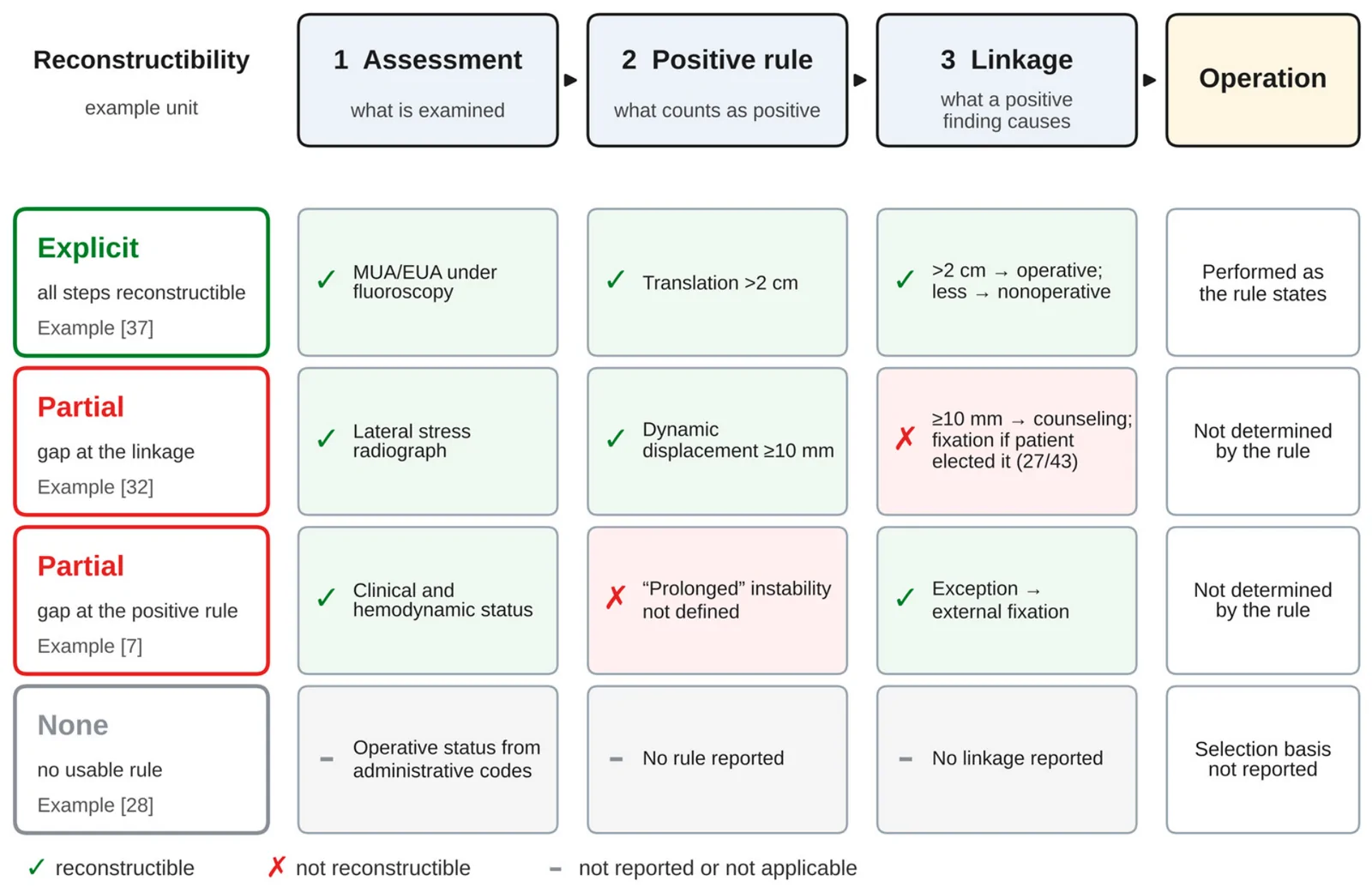
The operative-selection chain used to classify selection-rule reconstructibility, with one example from the included studies for each outcome. A unit was *Explicit* only when the assessment, the rule defining a positive finding, and the linkage from that finding to the operation could all be reconstructed; *Partial* when at least one could not; and *None* when no operationally usable rule could be reconstructed. Examples: *Explicit*, manipulation or examination under anesthesia with >2 cm translation leading to operative treatment [37]; *Partial* at the linkage, a lateral stress radiograph threshold of ≥10 mm leading to counseling, with fixation if the patient elected it [32]; *Partial* at the positive rule, external fixation reserved for “prolonged” hemodynamic instability without a stated definition [7]; *None*, operative status identified from administrative codes without any reported selection basis [28]. Green, red, and grey shading corresponds to reconstructible, non-reconstructible, and not reported or not applicable steps, respectively, as indicated by the symbols in the legend.

Figure 2 shows the chain on which this classification rests—assessment, positive rule, linkage to operation—with one example from the included studies for each outcome. Only six of 29 study-level units (20.7%) met the prespecified definition of *Explicit*, meaning that the assessment or variable, a positive threshold or clear decision rule, and the linkage to actual operative treatment could all be reconstructed. Twenty units (69.0%) were classified as *Partial*, most commonly because the final treatment decision remained individualized or discretionary, or because one or more components of the decision pathway were incompletely reported. Three units (10.3%) were classified as *None*, indicating that no operationally usable operative-selection rule could be reconstructed.

In a post hoc decomposition of the 23 units not classified as *Explicit*, two, by design, had no ascertainable clinical operative-selection pathway: one was an administrative claims-code analysis and one allocated treatment by randomization or patient preference. The remaining 21 units comprised the 20 *Partial* units and one *None* unit in which treatment had been left to surgeon discretion within an ordinary clinical pathway. Among these 21 units, the assessment or candidate variable was non-reconstructible in only two, whereas the rule defining a positive finding was non-reconstructible in 14 and the linkage from that finding to operative treatment in 16. In every one of these 21 units the non-reconstructible element lay at the definition of a positive finding, at its linkage to operative treatment, or at both—at both in nine. Both units in which the assessment itself was non-reconstructible also lacked a reconstructible positive rule.

The limitation in reconstructibility was therefore not the absence of a selection concept. In 19 of the 21 units, the assessment itself was reconstructible, and the failure occurred downstream of it. Because the terminal-step rule classifies a pathway ending in counseling, shared decision-making, or individualized judgment as *Partial* even when it is fully described (Section 2.6), these counts combine incompletely reported pathways with pathways that were reported clearly but were non-deterministic by design; the proportion classified as *Explicit* is the frequency of pathways meeting the prespecified definition, not a rate of adequate reporting.

### 3.7. Sensitivity Analyses for Participant Overlap and Denominator Composition

The primary synthesis retained the eight-center multicenter series as a separate study-level unit because it represented a distinct multicenter analysis, although participant overlap with selected earlier institutional series was considered highly probable but unquantifiable. Excluding that study reduced the denominator from 29 to 28 study-level units.

The overall pattern was unchanged. *Mixed* indications remained the most frequent category (14/28, 50.0%). Selection-rule reconstructibility was *Explicit* in 6/28 units (21.4%), *Partial* in 19/28 (67.9%), and *None* in 3/28 (10.7%). Thus, the principal descriptive findings were not materially dependent on inclusion of the multicenter cohort.

Two further post hoc sensitivity analyses addressed the composition of the denominator. Collapsing four units drawn from a single institutional registry into one cluster left 26 units, and restricting the denominator to units in which a clinical operative-selection pathway was applicable by design left 27; applying both left 24. *Explicit* remained six units in every scenario, corresponding to 20.7%, 23.1%, 22.2% and 25.0% respectively. Under the applicability restriction, two of the three units classified as *None* were removed, because both reflected designs in which no clinical operative-selection pathway existed rather than a failure to report one; the third, in which treatment was left to surgeon discretion, was retained.

### 3.8. Reproducibility of the Coding Framework

In post hoc independent recoding of the 29 study-level units—the 24 included at submission and the five added during peer review—with the second coder blinded to the original coding and analysis outputs, percent agreement was 89.7% (26/29; 95% CI 73.6–96.4) for selection-rule reconstructibility, 82.8% (24/29; 65.5–92.4) for indication category, and 96.6% (28/29; 82.8–99.4) for the presence of a quantitative threshold; corresponding κ values were 0.80 (95% CI 0.56–1.00), 0.72 (0.50–0.93) and 0.93 (0.73–1.00). The two coders agreed on all three fields in each of the five added units. In the 24 units included at submission, for which the analysis plan was fixed, agreement was 87.5% (21/24; 69.0–95.7), 79.2% (19/24; 59.5–90.8) and 95.8% (23/24; 79.8–99.3) respectively (κ 0.76, 0.64 and 0.91). Agreement in the 24 units remaining after exclusion of five units potentially exposed by the codebook’s worked examples was 91.7%, 79.2% and 95.8% respectively (κ 0.85, 0.69 and 0.91).

The nine field-level disagreements—five in indication category, three in selection-rule reconstructibility, and one in quantitative-threshold presence—occurred across seven of the 24 study-level units included at submission; none occurred in the five added units. All were classified as Type B—interpretive disagreements possible within the locked coding rules rather than misapplication of the codebook or errors of extraction (Section 2.7)—so the original coding was retained in every case, and no coded value was changed. Two units accounted for disagreements about whether a selection basis had been reported at all or reported incompletely. In a sensitivity analysis applying the second coder’s classification to both units, *Explicit* remained six of 29 units (20.7%), and among the resulting 18 partially reconstructible units the positive rule was non-reconstructible in 11 and the criterion-to-treatment linkage in 14, with every unit affected at one or both steps; the assessment itself was non-reconstructible in none. The proportion classified as *None* rose from three to five, of which two reflected designs in which no clinical operative-selection pathway could be ascertained. These two units were carried through the component decomposition because they alone move a unit across the *Partial*–*None* boundary, which determines whether a unit is treated as having no operationally usable rule rather than an incompletely reconstructible one. Had the second coder’s codes instead been adopted for all 29 units, including the single unit the second coder classified as *Explicit* rather than *Partial*, the distribution would have been *Explicit* in 7 units (24.1%), *Partial* in 17 (58.6%), and *None* in 5 (17.2%). All nine disagreements, with the supporting text cited by the second coder, are listed in Table S1. Table 3 sets the principal results under the original coding beside those obtained under the second coder’s codes; under either coding, a fully reconstructible operative-selection rule was the exception, and every partially reconstructible unit failed at the positive rule, the linkage to operation, or both.

## 4. Discussion

### 4.1. Principal Findings

This review examined how existing clinical studies reported the criteria used to select patients with LC-1 pelvic ring injuries for operative treatment. Across 29 study-level units representing 37 eligible reports, operative selection was described through a broad and heterogeneous set of criteria rather than a single reproducible decision rule. Just over half of the studies (15/29, 51.7%) used mixed selection pathways, and more than half (16/29, 55.2%) incorporated some form of dynamic or stress assessment. A quantitative threshold was reported in 18 studies (62.1%), yet only six (20.7%) provided a fully reconstructible pathway linking the assessment, a positive rule, and the actual decision to operate.

The nature of this shortfall in reconstructibility deserves precise statement. *Unspecified* was retained as a prespecified category for studies that invoked instability without describing how it was determined, yet no study was assigned to it. Almost every study described something about why patients underwent surgery, and the component decomposition locates the failure downstream of that description: among the 21 units with an applicable but incompletely reconstructible pathway, the assessment was identifiable in all but two, whereas the rule defining a positive finding failed in 14 and its linkage to the operation in 16, with every unit failing at one step or the other. The shortfall is therefore one of operationalization rather than silence—whether because a step was not reported or because the pathway was deliberately left to judgment—and it is not confined to the final link, since defining what counts as a positive finding failed almost as often as connecting that finding to surgery.

### 4.2. Static, Dynamic, and Clinical Decision-Making

Dynamic or stress assessment was the most common assessment construct identified in this review. Examination or manipulation under anesthesia and/or dynamic fluoroscopy was used in ten studies (34.5%), awake stress radiography in seven (24.1%), and stress ultrasonography in two (6.9%). This reflects the clinical concern that mechanically relevant instability may not be apparent on static imaging alone, prompting the development of dynamic assessment methods and awake alternatives to examination under anesthesia, including the lateral stress radiograph introduced by Parry et al. [2].

Dynamic testing has not, however, displaced clinical judgment; it has been layered onto it. Static morphology or displacement still contributed to selection in twelve studies (41.4%), and mobilization or functional clinical assessment in nine (31.0%). More tellingly, clinical failure repeatedly acted as a parallel or overriding pathway even inside studies designed to establish stress testing. In one prospective emergency-department stress cohort [3], two patients whose stress examination and repeat examination under anesthesia were both negative nevertheless underwent posterior fixation for intolerable pain preventing ambulation. A test intended to identify who needs surgery did not, in that study, determine who received it.

### 4.3. A Quantitative Cutoff Is Not a Validated Decision Rule

A central distinction emerging from this review is between a stated quantitative cutoff and a validated decision rule. Several influential studies stated a clear numerical criterion, yet their authors acknowledged limitations in the evidentiary basis of the threshold itself.

Displacement of approximately 1 cm (10 mm) was the most recurrent quantitative criterion, appearing in ten studies (34.5%) across emergency-department stress radiography, lateral stress radiography, and examination under anesthesia. Its apparent consistency should not be read as convergent validation, for three reasons.

**First, the same number denoted different measurements.** Approximately 10 mm was applied to *dynamic stress displacement* in ten studies, but in three to *static initial displacement* as a general operative threshold or pathway boundary. Identical figures did not describe identical constructs.

**Second, the value has a traceable lineage, its evidential basis is narrow, and several authors acknowledged this—although not in identical terms.** Sagi et al. [1] acknowledged that their 1 and 2 cm thresholds lacked a scientific basis. The same >1 cm criterion on examination under anesthesia was then built into a sequential fixation algorithm by an overlapping group of investigators [47]. DeKeyser et al. [3] subsequently adopted a 10 mm cutoff on emergency-department stress radiography but noted that this value might not have adequate support in the literature. Shawky et al. [40] again used a 1 cm threshold during sequential examination under anesthesia and explicitly described the cutoff as arbitrary. A separate study using a 2 cm manipulation threshold likewise described its treatment cutoff as arbitrary [37].

These acknowledgments should not be collapsed into a single claim: Sagi, Shawky, and Tosounidis presented their cutoffs as arbitrary or without a scientific basis, whereas DeKeyser described the adopted 10 mm value as potentially insufficiently supported by the literature.

One study did derive the value empirically. Within the Denver registry, Parry et al. [22] constructed a receiver operating characteristic curve for failure of nonoperative management—delayed fixation for pain-limited mobilization—against lateral-stress-radiograph displacement in 28 initially nonoperative patients; the area under the curve was 0.87, and 10 mm maximized sensitivity (100%) and specificity (78%). This is the only data-driven derivation of the threshold identified in the review, and it should be weighed alongside the lineage described above. Its evidential weight is nevertheless limited. The cutoff was derived from ten events at a single institution and has not been externally validated, and the outcome against which it was derived was itself shaped by the threshold: as the authors acknowledged, the treating surgeons were already routinely using 10 mm of displacement to guide decision-making, so patients below it were less likely to be offered delayed fixation. A threshold already in use cannot be independently validated against decisions it helped to make.

Read together, these reports suggest that recurrence of the 1 cm threshold over fifteen years reflects, at least in part, propagation of an established convention supported by a single internal derivation, rather than repeated independent validation of a clinically derived decision boundary.

**Third, and most fundamentally, the input stimulus that produces the measured displacement has not been standardized.** In the early examination-under-anesthesia literature the magnitude of manually applied force was not quantified. DeKeyser et al. [3] likewise reported that no standard quantified force was applied during either emergency-department stress examination or EUA and emphasized that *“there is currently no evidence in the literature to guide how much force should be applied in a pelvic stress examination.”* Parry et al. [2], in introducing the lateral stress radiograph, identified the same problem in the method they sought to replace, listing among the disadvantages of examination under anesthesia *“the subjective amount of force used by the examiners.”* Their alternative was explicitly designed around this problem: the lateral stress radiograph uses the patient’s own body weight in the lateral decubitus position to standardize the source of the deforming load. The method is therefore distinctive in constraining the maneuver itself rather than relying on an unmeasured manual force.

Direct measurement has since confirmed the underlying concern. Lee et al. [48] instrumented simulated examination under anesthesia on cadaveric specimens with a handheld dynamometer across eighteen orthopaedic traumatologists. Surgeon-level mean applied forces ranged from approximately 40 to 368 N in internal rotation, 0 to 317 N in external rotation, and 135 to 252 N in push-pull; the 0-N external-rotation value reflected a surgeon who used gravity alone in the frog-leg position. Variability *within* a single surgeon repeating the same maneuver was also substantial and maneuver-dependent: 3 of 17 surgeons (18%) varied by more than 50 N on repeated internal rotation and 1 of 17 (6%) on external rotation, whereas 6 of 7 (86%) did so on push-pull, with within-surgeon ranges reaching approximately 193 N. Lee et al. therefore suggested standardization through bodyweight stress or direct force measurement with dynamometers, approaches that address the stimulus itself rather than only the displacement measured after it is applied.

A reported displacement of 10 mm therefore cannot be assumed to represent the same mechanical challenge across studies when the applied force, its direction, patient position, imaging projection, and measurement technique all vary. The repeat-trial data further show that variation can persist even when the same surgeon repeats the same maneuver.

This concern is not new. In an invited commentary accompanying the early EUA threshold literature, Reilly [49] cautioned that reclassification based on intraoperative displacement was premature without standardization of the stress evaluation and clinical correlation of the measured instability, while recognizing the potential value of stress testing for revealing occult instability. More than a decade later, the present review finds the same recurrent displacement threshold applied across stress techniques whose mechanical stimulus remains incompletely standardized.

Accordingly, reproducibility of a displacement threshold depends on reproducibility of the mechanical stimulus used to generate that displacement.

Finally, even where a threshold was met, its consequence varied. In several studies, exceeding the threshold triggered counseling, operative consideration, or shared decision-making rather than fixation. The observed proportions make this concrete: in one stress-positive cohort [32], 27 of 43 patients proceeded to fixation after counseling on the risks and benefits of surgery, while the remaining 16 elected nonoperative management; in a registry-based series [20], 52 of 80 patients above the dynamic threshold underwent fixation compared with 1 of 48 below it. A threshold that identifies a population is not the same as a rule that determines an operation, and much of the *Partial* classification in this review arises from exactly this gap.

A multicenter study published after our search date illustrates both points [50]. Drawing on the same eight-center consortium as the multicenter series included here [17], and therefore probably overlapping with it, Achebe et al. restricted their cohort to LC-1 injuries of intermediate radiographic severity (Beckmann score 7–9) with a documented stress examination and compared operative with nonoperative management across stress-displacement strata of less than 12 mm, 12–16 mm, and more than 16 mm. Operative management was associated with fewer early adverse or ambulatory-decline outcomes in the 12–16 mm stratum, which comprised 31 patients; the authors described the finding as statistically fragile and hypothesis-generating. The study used neither the 10 mm convention nor a single operative threshold, and its population was itself conditioned on a radiographic severity score, so it reinforces rather than resolves the heterogeneity in how operative candidates are defined. Because it was published after the search date, it was not eligible for inclusion and contributes to no count reported here.

### 4.4. Layers of Variability in LC-1 Decision-Making

The measurement problem sits within a broader and well-documented hierarchy of variability that can be considered at three distinct levels.

At the level of classification, Furey et al. [9] had five orthopaedic trauma surgeons classify 89 pelvic ring disruptions using both the Young–Burgess and the Tile systems on two occasions and found only a moderate degree of agreement with either system, concluding that the observed inter- and intraobserver variability may limit the usefulness of the two classifications. Variability therefore arises before any treatment threshold is considered.

At the level of the operative decision, Beckmann et al. [10] presented 27 LC-1 cases—each with anteroposterior, inlet and outlet radiographs and a scrollable computed tomography series—to 111 Orthopaedic Trauma Association members. Overall agreement on whether to operate was fair (κ = 0.39; 95% CI, 0.34–0.44), and only 9 of the 27 cases (33%) reached substantial agreement. The distribution of responses was also uneven: in four cases nearly every respondent chose fixation (93.1–94.4%), whereas in five almost none did (0–8.7%). This pattern suggests that disagreement was not uniform across the case spectrum and was greatest away from the most obvious treatment extremes—a clinically relevant observation for the minimally displaced injuries in which threshold-based decision rules are most often debated. Years in practice, annual pelvic fracture volume, and completion of a trauma fellowship were not significant predictors.

At the level of the stress test itself, Carney et al. [51] distributed ten pelvic-injury vignettes to fifteen experienced pelvic surgeons, defining agreement as more than 80% similar responses. Surgeons agreed on whether the ring was stable or unstable in 8 of 10 cases, but agreed on whether fixation was required in only 6 of 10; the authors concluded that surgeons generally agreed on whether an EUA was positive or negative but not necessarily on the implications of that result. Practice diverged at the level of the examination as well: 7 of 15 (46.7%) performed a full 15-part examination under anesthesia while the other 8 (53.3%) used an abbreviated or alternative method, and only 8 of 15 provided a definition of what constitutes a positive examination, the remaining 7 not endorsing adherence to a strict definition. A subsequent survey from the same group [6] put ten pelvic ring injury vignettes, again drawn from a single Level I trauma center, to eleven experienced pelvic surgeons, first with imaging alone and then with examination under anesthesia added. The examination clearly improved agreement about *stability*: agreement rose from 4 to 8 of 10 cases and interreviewer reliability from 0.207 to 0.592. Agreement about *the operation* improved much less: from 5 to 6 of 10 cases, with reliability rising from 0.250 to 0.432. Those authors isolated the point themselves: of the eight cases in which the surgeons agreed on stability, only six carried concordant agreement on the need for fixation.

The three levels point in the same direction: uncertainty exists in classification, in the procedure and interpretation of stress testing, and in the operative decision itself. Across the surveys of Carney et al. [51] and Tucker et al. [6]—which share authors and a single institutional source of cases, and are therefore not independent replications—providing or interpreting EUA information improved agreement about *stability* more than agreement about *the operation*; each found agreement on fixation in only 6 of 10 cases when EUA findings were available. These data suggest that improving measurement alone does not resolve the decision gap: a substantial part of the uncertainty lies in the translation from the measured finding to treatment. The present finding that only 6 of 29 studies (20.7%) described a selection pathway that was fully reconstructible under the prespecified definition is the published-record counterpart of that gap.

### 4.5. Implications for Treatment-Effect Synthesis

A previous systematic review [11] restricted its population to unstable non-fragility LC-1 injuries with complete sacral fractures, yet still identified only five observational studies, comprising 183 surgically and 314 nonsurgically treated patients. Formal meta-analysis was not possible because outcome time points and reporting were heterogeneous, and the authors concluded that there was no agreed protocol for surgical intervention in this heterogeneous injury pattern.

More importantly for the present review, Varma et al. identified variable eligibility criteria for surgery across the included studies: some emphasized pain and mobility, whereas others emphasized initial displacement on static radiographs. They also noted persistent uncertainty in the definition of instability itself, which could be framed either as a tendency to displace or as excessive pain with mobilization despite maintained bone position. What that review encountered as an obstacle to pooling within a narrowly defined population, the present review takes as its primary object and characterizes across a wider treatment-selection literature.

The fact that the earlier review restricted eligibility to complete sacral fractures highlights the broader challenge of assembling comparable populations. In the present review, several studies restricted enrollment by initial displacement, others by posterior-ring morphology, others to stress-positive injuries only, and one analyzed an operative-only cohort whose entry was conditioned on radiographic instability or failed mobilization [26]. When the construct defining an operative candidate differs across studies—and when meeting a criterion may lead to fixation, counseling, or neither—pooled operative-versus-nonoperative estimates may compare populations that were constituted differently before outcomes were measured.

### 4.6. Operative Selection as a Preference-Sensitive Decision

The predominance of mixed pathways also suggests that operative selection should not be reduced to mechanical displacement alone. Pain, ability to mobilize, fracture morphology, associated injuries, surgeon judgment, and patient preference repeatedly modified or overrode imaging-based criteria in the included literature.

This is clinically plausible, because the decision to operate is intended to improve outcomes that matter to patients rather than to normalize a stress image. In a discrete-choice experiment involving patients with LC1 pelvis fractures, Patterson et al. [52] found that respondents placed greater value on early independent ambulation, discharge home, reduced pain, shorter hospitalization, and avoidance of secondary surgery than on operative versus nonoperative treatment itself, with no significant intrinsic preference for either modality.

Accordingly, the incompleteness of a purely mechanical rule may not represent methodological weakness alone; it may also reflect the inherently preference-sensitive nature of this treatment decision. This does not, however, justify poorly specified decision pathways. Shared decision-making is not the problem; failing to report which objective finding led to counseling, and which clinical or patient-level factors then determined the operation, is.

### 4.7. What Future Studies Should Report

The practical implication is that operative-selection reporting requires the rigor customarily applied to outcome reporting. Based on the recurrent reporting gaps identified in this review, we suggest several pragmatic reporting elements for future studies; these are not intended as a formally validated reporting checklist. Future studies should separate three distinct questions:

(1) how mechanical instability is elicited, (2) how a positive test is defined, and (3) how that result is linked to the decision to operate.

At minimum, reports of dynamic assessment should specify:

1. **Patient position** and whether the stimulus was surgeon-applied, patient-generated, or a defined external load;
2. **The stress maneuver**, including direction, and the magnitude of applied force or load where measurable, together with who performed the examination;
3. **Imaging projection and measurement landmarks**, and whether a calibrated reference was used;
4. **The definition of displacement** and the positive threshold stated exactly, including the direction of the inequality and how boundary values were handled;
5. **What a positive result triggered**—mandatory fixation, an operative recommendation, counseling, or consideration only;
6. **How many threshold-positive patients actually underwent surgery**, and how many threshold-negative patients were converted, with reasons;
7. **Population restrictions conditioning the denominator**, stated separately from the operative threshold so the two are not conflated;
8. **Clinical override and conversion criteria**—pain-limited mobilization, secondary displacement, associated injuries, patient preference—reported explicitly rather than subsumed under a generic statement of “instability.”

Items 5 and 6 are particularly consequential because they determine whether a reported criterion can be linked to the treatment actually delivered. Reporting a threshold without reporting what it caused leaves the reader unable to distinguish a decision rule from a descriptive cutoff.

### 4.8. Limitations

**Purpose and scope.** This review characterizes how operative-selection criteria have been reported; it does not establish the optimal indication for fixation and does not compare the effectiveness of operative and nonoperative treatment. It sampled the treatment-selection literature rather than all LC-1 clinical literature, and the proportions reported here should not be read as reporting rates for the LC-1 literature as a whole.

**Structural asymmetry of the sampling frame.** Mixed pelvic-ring cohorts were eligible only when LC-1-specific treatment-selection information could be separately extracted. *Mixed-injury cohorts may therefore be enriched for better LC-1-specific reporting because separately extractable selection information was itself required for eligibility*, and the *Not reported* category can arise only in studies whose primary analytic population was explicitly defined as LC-1. The distribution of selection-rule reconstructibility should be interpreted with this asymmetry in mind.

**Heterogeneity of the included study designs.** The 29 study-level units span more than three decades and include a 1990 protocol-based observational cohort, retrospective and prospective institutional series, diagnostic stress-imaging studies, a prospective clinical trial with randomized and preference arms, and a national administrative database analysis. Sample sizes ranged from tens of patients to a coded cohort of more than seventy thousand, and definitions of LC-1, imaging protocols, and treatment eras differ correspondingly. Studies within a single indication category are therefore not methodologically comparable with one another, and the proportions reported here describe the distribution of reporting practice across a heterogeneous body of work rather than an estimate derived from a homogeneous set of studies. Treatment era includes the COVID-19 pandemic. Enrollment period was not a prespecified extraction field, but on post hoc review of the source reports, at least nine units [20,26,27,32,33,35,40,42,43] had enrollment periods overlapping 2020–2022, when constrained operating-room, critical-care, and inpatient resources altered pelvic trauma care, including hospital stay and transfusion practice [53]. Selection pathways reported from those cohorts may therefore partly reflect resource availability rather than stable institutional practice; the review was not designed to test this.

**Search scope.** Two databases were searched without language or publication-type filters, with English-language full text required for final eligibility, and supplemented by reference-list screening of the 31 reports included at submission, forward citation searching of six prespecified sentinel papers, and a final database update, none of which identified an additional eligible report; citation searching was not repeated for the six reports added during peer review. The strategy required lateral-compression or LC-1 terminology in searchable fields; therefore, otherwise eligible clinical studies describing LC-1-equivalent morphology without this terminology may have been missed. The search strategies did not undergo formal peer review using the PRESS guideline [54]. As a partial safeguard, they were tested against prespecified validation records: all five core records were retrieved by both final strategies, and all four boundary records by the PubMed strategy. Known-item retrieval tests sensitivity only for records already known to the reviewer and does not substitute for independent peer review of the strategies.

**Single-reviewer process.** Screening, extraction, and coding were performed by one human reviewer, supported by structured second-pass source verification, AI-assisted discrepancy detection and source cross-checking, and a dated amendment log rather than duplicate independent human review. Exclusions were first proposed by a generative-AI system: at title screening they were audited rather than individually re-adjudicated, and at abstract screening each was confirmed by that reviewer. A title-level error outside the audited subset, or an error shared by a proposal and its confirmation, would not have been detected, and the 297 records excluded from the supplementary searches were not re-checked. The blinded sample check by a second reviewer (Section 2.4) reduces but does not remove this concern: it covered 30 of 374 exclusions, and the second reviewer was by then a co-author aware of the final set of included studies. The blinded sample check showed that this process was not free of error: two of 10 sampled abstract-level exclusions were eligible, and the subsequent re-screening of all abstract-level exclusions identified four more eligible reports, all of which have been added. Title-level exclusions were checked only by audit and by a sample of 20, none of which was disputed, so residual missed reports at that stage cannot be excluded. Classification of selection pathways involves judgment, and reviewer-dependent classification bias cannot be excluded.

**Reproducibility assessment.** The reproducibility assessment was conducted after the prespecified review rather than as prospective duplicate coding, involved a single additional coder who subsequently joined as an author, and was adjudicated by the original reviewer; with only 29 units, κ may change materially with a small number of disagreements. The five units added during peer review were recoded after the second coder had become an author. The codebook used by both coders contains worked examples from which the classification of certain studies could be inferred; agreement in the units remaining after exclusion of five units potentially exposed by those worked examples is reported alongside the primary figure. Agreement was lower for indication category than for selection-rule reconstructibility, and the disagreements clustered at two boundaries: whether a study reporting more than one determinant should be classified *Mixed*, and whether a selection basis had been reported at all or reported incompletely—the boundary the prespecified codebook created the *Unspecified* category (instability invoked without a stated means of determining it) to occupy, and to which no unit was assigned. The coding framework also contains no separate category for allocation by randomization, which produced one further disagreement. The second coder recoded the three prespecified fields only; the post hoc component decomposition that underpins the principal interpretation—that reconstruction failed at the positive rule and the linkage rather than at the assessment—was performed by the original reviewer alone and was not independently recoded. Table 3 applies that decomposition to the second coder’s classifications, but the component judgments themselves remain single-reviewer judgments.

**Category assignment and reporting granularity.** Category assignment depended on what each report described as a determinant of selection. The proportion of units classified *Mixed* may therefore reflect reporting granularity in addition to genuine clinical multifactoriality.

**Dependence on published description.** The review could only reconstruct decision-making that was described. Undocumented clinical reasoning was intentionally not inferred, so *Partial* and *None* classifications reflect what was reported and, for pathways ending in counseling or individualized judgment, the non-deterministic design of the pathway itself, rather than necessarily the quality of the underlying clinical decision. A *Partial* classification does not imply that shared decision-making was inappropriate; it indicates that the prespecified coding framework could not reconstruct a fully specified rule linking the reported finding to the operation performed.

**Design and coding.** This is a descriptive methodological review, so no formal risk-of-bias instrument or certainty-of-evidence assessment was applied, and the review outcome was deliberately not repurposed as a quality score (Section 2.8). Readers seeking a quality appraisal of the included studies will not find one here.

**Participant overlap.** Several included studies drew on overlapping institutional registries. Relationships that could not be established as the same, reused, or nested analytic cohort were flagged but retained as separate study-level units. A prespecified sensitivity analysis excluding the eight-center multicenter series [17] left the principal findings unchanged.

**Registration.** The review was registered on the Open Science Framework after screening and extraction had been completed. The governing plan and extraction codebook were locked before full extraction, and the locked documents, search strategies, dataset, and dated amendment record are publicly deposited, but the registration is retrospective and should be read as such.

## 5. Conclusions

Published operative indications for LC-1 pelvic ring injuries remain heterogeneous and incompletely operationalized. Displacement of approximately 1 cm is the most recurrent quantitative criterion, but repetition should not be mistaken for validation: key studies described the cutoff as arbitrary or as possibly lacking adequate literature support; its only data-driven derivation came from a small single-institution cohort in which the threshold was already guiding treatment; the same figure has been applied to different measurements; and the mechanical stimulus producing that displacement is itself not standardized. The published literature depicts operative selection as a multifactorial, preference-sensitive pathway integrating mechanical assessment with morphology, symptoms, mobilization, and patient priorities—which makes explicit reporting of the decision pathway more important, not less. More reproducible operative indications will require better standardization of stress assessment, explicit reporting of the linkage from test result to treatment, and prospective validation against outcomes that matter to patients.

## Data Availability

The prespecified review plan and extraction codebook, as locked on 12 August 2026 before extraction, together with the as-used search strategies, the protocol/data reconciliation record and the source-locked study-level dataset of 24 units (31 reports) locked before submission, are openly available in the frozen Open Science Framework registration at https://doi.org/10.17605/OSF.IO/WSDGX; the registration is not altered by later deposits. The analysis dataset as reported here (29 study-level units, 37 reports), the full audit and amendment archive, the records of the screening re-check performed during peer review, the second-coder reproducibility records, the PRISMA 2020 and PRISMA-S materials, and the manuscript with its supplementary tables are openly available on the linked OSF project at https://doi.org/10.17605/OSF.IO/SNQDZ. Apart from the additions to the registry unit described in Section 2.9, the coded values of the original 24 units are the same in both records.

## Supplementary Materials

The following supporting information can be downloaded at: https://www.mdpi.com/article/10.3390/jcm15207891/s1, Supplementary Methods S1, giving the procedural details of AI-assisted screening (model inputs, outputs, and logging), the second-coder calibration and blinding procedure, the bootstrap specification for κ, and the dated timeline of the review; Table S1, listing every field-level disagreement between the two coders with the supporting text cited by the second coder and its adjudication [4,7,19,27,31]; Table S2, listing the eight reports excluded after full-text assessment with their citations and reasons for exclusion [55,56,57,58,59,60,61,62]; the completed PRISMA 2020 checklist; the PRISMA-S search-reporting checklist; and the PRISMA 2020 flow diagram (Figure 1). The governing protocol and codebook, the as-used search strategies, the locked study-level dataset, the protocol/data reconciliation record, the supplementary search and reproducibility records, and the full audit archive, including the screening workbook with all AI proposals, are deposited on the Open Science Framework (see Data Availability Statement).
