# Supplementary material for: Operative Indications for Lateral Compression Type 1 Pelvic Ring Injuries: A Systematic Review of Reported Selection Criteria
Source: J Clin Med. 2026 Oct 13;15(20):7891. doi: 10.3390/jcm15207891 (PMC13705429; doi:10.3390/jcm15207891)
Supplement: Supplementary file 1 [file jcm-15-07891-s001.zip › Supplementary_Methods - S1.pdf]

Supplementary Methods S1

Operative Indications for Lateral Compression Type 1 Pelvic Ring Injuries: A Systematic Review of Reported Selection Criteria

This supplement gives the procedural detail summarized in Sections 2.4, 2.7, 2.8 and 2.9 of the main text. Sections S1.6 and S1.7 describe the checks of screening performed during peer review and the reports added as a result. All records referred to below are deposited on the Open Science Framework (registration https://doi.org/10.17605/OSF.IO/WSDGX; project https://doi.org/10.17605/OSF.IO/SNQDZ).

S1.1. Generative-AI assistance at title and abstract screening

System. ChatGPT (OpenAI; model/version GPT-5.6 Sol) was used for screening support. A second system, Claude (Anthropic; Claude Opus 5), was not used for screening; it was used after extraction for an independent source-verification pass and for workbook and package integrity auditing (main text, Section 2.7).

Stages at which AI proposals were used.

Stage	Records	Input supplied to the model	Output requested	Human step
Title screening	420 unique records	Bibliographic record (title, authors, journal, year, database publication type) and the locked screening codebook	Proposal: abstract review or exclusion; title-level code (E1–E5) and a short reason	Not individually re-adjudicated. Audited on 12 August 2026 (AI-assisted audit reviewed by H.K.S.): every prespecified sentinel record had been forwarded, and review of the 27 excluded titles combining lateral-compression and treatment or instability terms found no erroneous exclusion. The Human_TA_Decision field accordingly reads “Not required” for these records. Of 304 proposed exclusions, 297 remained exclusions, 4 were reclassified as linkage-only registry or protocol records, and 3 were retained as background literature
Abstract screening	116 records forwarded from title screening	Title, abstract and bibliographic metadata, with the locked screening codebook (codes P1–P5, D1–D10, X1–X4)	Proposal: *Include*, *Unclear* or *Exclude*; detailed code; reason; supporting abstract text	All 77 proposed exclusions reviewed and confirmed by the human reviewer, with 0 overrides; 35 *Include* or *Unclear* records advanced to full text; 4 linkage-only records. During peer review, 10 of the 77 exclusions were forwarded to full text by the blinded sample check and the subsequent re-screening (Sections S1.6 and S1.7), leaving 67 abstract-level exclusions
Full-text assessment, linkage, extraction	45 reports (35 at the original screening; 10 forwarded during peer review)	—	No AI proposals	Performed by the human reviewer
Re-screening of abstract-level exclu-	75 records	—	No AI proposals	Performed by the human reviewer without access to the original decisions (Section

Stage	Records	Input supplied to the model	Output requested	Human step
sions (peer review)				S1.7)

Abstract-level exclusions remaining after the peer-review checks (n = 67), by original code. Operative-only or fixation-construct outcome study without a selection criterion (D8), 15; clinical or imaging study without treatment-selection linkage, 11 (for these the assistant gave a reason but no code, and the code was assigned by the human reviewer); mixed population without separately extractable LC-1 information (P2), 10; conference abstract only (D7), 8; review article (D1), 8; non-English full text (X1), 5; dedicated fragility or low-energy geriatric cohort (P5), 5; case report (D6), 2; editorial or commentary (D3), 1; decision or analytic model (D5), 1; outside LC-1 (P1), 1. The 10 exclusions later forwarded to full text had carried the original codes D8 (2), no assistant code with a reviewer-assigned code (5), D4 (1), D7 (1) and P5 (1); their outcomes are given in Section S1.7. Before the peer-review checks, the 77 confirmed exclusions comprised D8 17, reviewer-coded 16, P2 10, D7 9, D1 8, P5 6, X1 5, D6 2, D4 1, D3 1, D5 1 and P1 1.

Logging. Each AI proposal was written to dedicated columns of the screening workbook (sheet TA_Screening_420: Assistant_Title_Triage, Assistant_E_Code, Assistant_Reason, Assistant_Abstract_Decision, Assistant_Abstract_Code, Assistant_Abstract_Reason, Assistant_Abstract_Evidence, Proposed_TA_Action), separately from the human decision and final record action (Human_TA_Decision, Human_Code, Record_Action). The human confirmation of the 77 abstract-level exclusions is recorded record by record in sheet AI_Exclude_Audit_77, and the confirmation was entered in the amendment ledger on 18 August 2026 (PLA-41). The workbook is deposited in the OSF full audit archive.

Records from supplementary searches. The 183 new records from reference-list checking and the 113 new records from forward citation searching were screened in the same way; all were excluded with human confirmation (0 overrides). The single new record from the database update was excluded by the human reviewer as a letter to the editor.

S1.2. Independent second-coder recoding

Analysis plan. A statistical analysis plan for the reproducibility assessment (version 1.3) was fixed before the second coder's entries were returned, and its SHA-256 checksum was recorded (amendment ledger PLA-42). The plan defined the fields to be compared, the agreement statistics, the sensitivity analysis for worked-example exposure, and the Type A/Type B classification of disagreements.

Blinding and materials. The second coder (J.L.) received the locked codebook, the source reports, and a coding sheet in which study identifiers had been relabeled and the order of units changed. The original coding, the locked dataset, the amendment record, the analysis outputs and the manuscript were withheld.

Calibration. Before coding the 24 analytic units, the second coder coded four reports that had been excluded at full text, to become familiar with the codebook. After calibration, no study-specific discussion or feedback was permitted; procedural questions could be raised, but the locked coding rules were not modified.

Fixing of entries. The completed coding sheet was returned on 18 August 2026, and its SHA-256 checksum was recorded before the crosswalk to the original identifiers and the original coding were opened (PLA-43).

Classification of disagreements. The original reviewer (H.K.S.) classified each disagreement after the second coder's entries had been fixed. Type A was defined as a clear misapplication of the codebook or an error of source extraction, to be corrected; Type B as an interpretive disagreement possible within the locked coding rules, for which the original coding was retained and the disagreement reported. No consensus meeting was held, and the second coder took no part in the classification (PLA-44). All nine field-level disagreements were classified as Type B (Table S1).

Recoding of the five units added during peer review. The five study-level units added during peer review (Section S1.7) were recoded by the same second coder under the same procedure: the coding sheet carried relabeled identifiers (N01–N05) in an order set by a fixed seed (20261007, an arbitrary integer), the locked codebook was unchanged, and the original coding, the key, the manuscript and the decision records were withheld. No further calibration was performed. By this time the second coder was a co-author. The returned sheet was fixed with its SHA-256 checksum on 6 October 2026 before the key was opened. The two coders agreed on all three fields in all five units, so no disagreement required classification.

S1.3. Agreement statistics

Percent agreement was calculated with Wilson 95% confidence intervals. Cohen's κ was unweighted. Its 95% confidence intervals were obtained by bootstrap resampling of the study-level units with replacement, with 2000 replicates per analysis, taking the 2.5th and 97.5th percentiles of the resulting κ distribution. Replicates in which κ was undefined were discarded; this occurred once, in a pre-revision analysis of threshold presence (19 units) that is not reported in the manuscript, and in none of the analyses reported in the manuscript. Calculations were performed in Python 3.11 using the standard-library Mersenne Twister generator, re-initialised with the fixed seed 20260814 before each analysis. All statistics were computed on pre-adjudication codes. After the five units added during peer review had been recoded, the same statistics were computed, with the same procedure and seed, for the 29 units of the final review and for the 24 of them not potentially exposed by the codebook's worked examples; the figures for the 24 units covered by the analysis plan are reported alongside.

S1.4. Dated timeline of the review

Date	Step	Record
12 August 2026	Review plan and 11-field extraction codebook locked (version 2.2)	Governing plan, OSF registration
12 August 2026	PubMed (350) and Embase (401) searches executed; deduplication 751 → 420	Search-strategy file; screening workbook
12 August 2026	Title screening (304 proposed exclusions; audit by sentinel check and 27-title re-review) and abstract screening (116 records; 77 proposed exclusions; 35 advanced to full text)	Screening workbook
13 August 2026	Full-text assessment of 35 reports (4 excluded); report linkage (31 reports → 24 study-level units); data extraction; initial data lock	Extraction workbook
13–14 August	Structured re-verification, AI-assisted source-verification pass with human	Amendment ledger; reconciliation rec-

Date	Step	Record
2026	re-checking, post-lock amendments PLA-01 to PLA-40; final data lock	ord
14 August 2026	OSF registration (DOI 10.17605/OSF.IO/WSDGX)	OSF Registries
14 August 2026	Supplementary searches: reference lists of all 31 eligible reports, forward citation searching of six sentinel papers, database update; no additional eligible report	Search-closure record
18 August 2026	Human confirmation of the 77 assistant-proposed abstract-level exclusions entered in the ledger (PLA-41)	AI_Exclude_Audit_77; ledger
18 August 2026	Reproducibility analysis plan fixed (PLA-42); second coder's recoding returned and fixed (PLA-43); disagreements classified (PLA-44)	Reproducibility records
18 August 2026	OSF project deposit updated; manuscript submitted	OSF project
2 October 2026	Blinded second-reviewer check of screening exclusions: first packet (60 records) withdrawn without analysis; second packet (35 records, seed 20261012) fixed and sent (Section S1.6)	Sample-check plan v1.1; packets; keys
6 October 2026	Second reviewer's sample sheet returned and fixed with its checksum, then compared with the key; full-text assessment and extraction of the two forwarded exclusions by the original reviewer	Sample-check result sheet; decision record
6 October 2026	Re-screening of the 75 remaining abstract-level exclusions by the original reviewer; full-text assessment of the 8 forwarded reports (4 eligible, 4 excluded); extraction; report linkage (37 reports → 29 study-level units) (Section S1.7)	Re-screening sheet; full-text decision and extraction sheets; decision record
6 October 2026	Second coder's recoding of the five added units returned and fixed with its checksum, then compared with the key (Section S1.2)	Reproducibility records

S1.5. Non-English records

Nineteen of the 420 unique records were non-English publications, identified by the translated-title notation of their bibliographic records. Thirteen were excluded at title screening on content grounds (wrong population or clinical question, or excluded publication type). Six were excluded at abstract screening: five because only non-English full text was available (code X1) and one because LC-1-specific information was not separately extractable (code P2). No non-English report reached full-text assessment.

S1.6. Blinded second-reviewer check of screening exclusions (added at revision)

Design. In response to peer review, a random sample of exclusions was drawn with a fixed seed (20261012, an arbitrary integer set before the draw): 20 of the records excluded at title screening and 10 of those excluded at abstract screening. Records included in the withdrawn first packet (see below) were removed before the draw, so the sampling frame comprised 267 of the 297 title-level exclusions, 57 of the 77 abstract-level exclusions, and 25 of the 35 records advanced to full text; the sample is therefore a random sample of that frame rather than of all exclusions. To prevent the second reviewer from inferring that every record was an exclusion, five records advanced to full text were added, and the 35 records were presented in random order without their original decisions.

Procedure. The second reviewer (J.L.) screened each record at title-and-abstract level against the eligibility criteria and the screening codebook, recording *Exclude* (with a code) or *Forward* (possi-

bly eligible or uncertain), with a one-line reason drawn from the title or abstract for every record. He was instructed not to consult the original decisions, the audit workbook, the OSF records, or any AI tool. He was, however, a co-author by this time and aware of the final set of included studies. The completed sheet was fixed with its SHA-256 checksum before it was compared with the key.

Analysis. The primary measure was the number of the 30 excluded records that the second reviewer would have forwarded. Each such record was assessed at full text by the original reviewer against the locked eligibility criteria, and any record found eligible would be added to the review. Agreement on the five advanced records is reported as a secondary measure.

Earlier packet. A first packet (60 records, seed 20261002) was withdrawn without analysis: the returned sheet reproduced the original assistant codes, including codes not offered in the packet, and so could not be regarded as an independent assessment. Its records were excluded from the second draw. Both packets, their keys and the returned sheets are retained.

Result. The returned sheet met the prespecified acceptance conditions: a reason drawn from the title or abstract was recorded for every record, and the codes did not reproduce the original assistant codes. The second reviewer would have forwarded none of the 20 title-level exclusions, two of the 10 abstract-level exclusions, and all five advanced records. The two forwarded exclusions were assessed at full text by the original reviewer against the locked eligibility criteria, without AI proposals, and both were judged eligible: Abdelrahman et al. [43], an operative cohort reporting the institutional pathway for selecting mobilization, posterior fixation and added anterior fixation (sampling-frame conditions 2 and 3), and Brostowitz et al. [44], a cohort with operative and non-operative groups in which treatment was assigned at the attending surgeon's discretion (condition 1, coded as having no reported selection criterion). Both had been excluded at abstract screening on assistant-proposed or reviewer-assigned codes (D8 and a reviewer-assigned code, respectively) that the full text did not support.

Because two of 10 sampled abstract-level exclusions were in error, the error rate at that stage could not be regarded as negligible, and the remaining abstract-level exclusions were re-screened in full (Section S1.7). Because none of the 20 sampled title-level exclusions was disputed, title-level exclusions were not re-screened; with no disagreement among 20 title-level exclusions, the upper 95% bound of the proportion of erroneous title-level exclusions in the sampled frame is approximately 15% (rule of three); this bound does not cover the 30 title-level exclusions removed with the first packet, and residual missed reports at that stage cannot be excluded.

S1.7. Re-screening of abstract-level exclusions and reports added during peer review

Re-screening. The original reviewer (H.K.S.) re-screened the 75 original abstract-level exclusions other than the two found eligible in the sample check (that is, including the eight sampled exclusions the second reviewer had not disputed) against the eligibility criteria, at title-and-abstract level, without AI proposals and without access to the original decisions or codes. Records that

might be eligible, or for which eligibility was uncertain, were forwarded to full text. Eight were forwarded.

Full-text assessment. The eight forwarded reports were assessed at full text by the original reviewer against the locked eligibility criteria, without AI proposals. Four were eligible: Beckmann et al. [45], a radiographic score linked to operative or nonoperative recommendation; Lawson et al. [46], a nonoperative series reporting the acute treatment algorithm and the postmobilization stability assessment; Avilucea et al. [47], a sequential examination-under-anesthesia fixation algorithm; and Hadeed et al. [24], a registry report stating the departmental protocol by which static displacement of 10 mm or more led to fixation without examination under anesthesia. Four were excluded, as listed in Table S2: one on code P2, two as outside the treatment-selection sampling frame, and one on code P5.

Linkage and extraction. Linkage followed the locked rule (Manuscript Section 2.5). Hadeed et al. [24] was drawn from the same institutional lateral-stress-radiograph registry as an existing unit and was linked to it as a companion report; the additional pathway it reports (static displacement of 10 mm or more leading to fixation) was added to that unit's operative-selection, assessment-method and threshold entries, and the unit's indication category and selection-rule reconstructibility were unchanged. Avilucea et al. [47] shares investigators and the examination-under-anesthesia framework with an included unit, but the two cohorts differ in period, participating centers and examination result, and a same, reused or nested cohort could not be established; it was therefore kept as a separate unit and the possible overlap was flagged. Lawson et al. [46] came from a center that participated in the included multicentre series and was likewise kept separate with an overlap flag. The other three reports formed new units. Extraction of all 11 fields was performed by the original reviewer without AI proposals, using the locked codebook (version 2.2) without modification. In total, 10 reports were assessed at full text during peer review, six were added, and the review comprises 37 eligible reports and 29 study-level units, with eight companion reports and eight flagged relationships of possible partial overlap.

Effect on the analysis. All counts, proportions, sensitivity analyses and the component decomposition in the revised manuscript were recalculated on the 29 units. No eligibility criterion or coding rule was changed, and apart from the additions to the registry unit described above, no coded value of the original 24 units was changed.
